# Charge transfer dynamics in noble gas endofullerenes: intra- and extramolecular tunnelling

**DOI:** 10.1039/d5na00727e

**Published:** 2025-10-10

**Authors:** Ali Sufyan, Tyler James, Connor Fields, Shabnam Naseri, Filipe Junqueira, Sofia Alonso-Perez, Sally Bloodworth, Gabriela Hoffman, Mark C. Walkey, Elizabeth S. Marsden, Richard J. Whitby, Yitao Wang, David A. Duncan, Tien-Lin Lee, James N. O'Shea, J. Andreas Larsson, Brian Kiraly, Philip Moriarty

**Affiliations:** a Applied Physics, Division of Materials Science, Department of Engineering Sciences and Mathematics, Luleå University of Technology 97187 Luleå Sweden; b School of Physics & Astronomy, University of Nottingham Nottingham NG7 2RD UK philip.moriarty@nottingham.ac.uk; c School of Chemistry and Chemical Engineering, University of Southampton Southampton UK; d Diamond Light Source Harwell Science & Innovation Campus Didcot UK; e School of Chemistry, University of Nottingham Nottingham NG7 2RD UK; f Wallenberg Initiative Materials Science for Sustainability, Luleå University of Technology Luleå SE-97187 Sweden

## Abstract

Core-level and tunnelling spectroscopies applied to noble gas endofullerenes offer complementary insights into electron transfer rates, addressing both intramolecular and extramolecular processes. Elastic and inelastic tunnelling spectroscopy of empty C_60_ and Kr@C_60_ on Pb/Cu(111) each show that the encapsulated atom is essentially invisible to scanning probes. We interpret the lineshape of the lowest unoccupied molecular orbital (LUMO) of Pb-adsorbed (endo)fullerenes in tunnelling spectra as a signature of the dynamic Jahn–Teller (DJ–T) effect. This effect persists in electronically decoupled second-layer molecules, which also display distinct vibronic progressions in on-resonance tunnelling. DFT calculations reproduce the LUMO alignment and low density of states at the Fermi level seen in experimental tunnelling spectra for (endo)fullerenes on Pb, and, in line with submolecular resolution STM images, also predict that an atom-down orientation of the fullerene cage is energetically most favourable (although other adsorption geometries differ only by tens of meV at most). In contrast to the tunnelling data, core-level-focussed techniques – namely, photoemission, X-ray absorption, and resonant Auger–Meitner electron spectroscopy – of Ar@C_60_/Pb(111) indicate that the encapsulated atom is heavily coupled to the molecular environment, with both a clear influence of substrate screening on the core-level lineshape and the absence of spectator signal in decay spectra.

## Abstract 2

Core-level and tunnelling spectroscopies applied to noble gas endofullerenes offer complementary insights into electron transfer rates, addressing both intramolecular and extramolecular processes.

## Graphical abstract

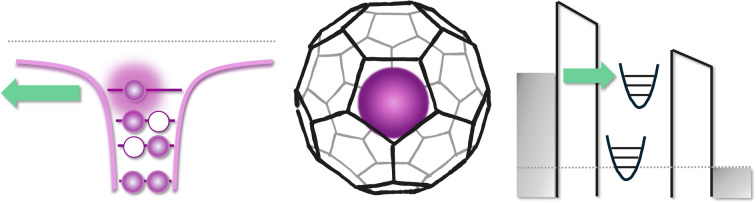

## 1 Introduction

“All chemistry is femtochemistry.” Although sometimes misattributed to Zewail,^1^ it was Donoso and Martens^2^ who coined this memorable aphorism (in 1998), on the following basis: “*…the underlying elementary dynamical steps* [in chemical reactions] *occur on the ultrafast time scales of molecular translations, vibrations, rotations, and electronic transitions, most conveniently measured in femtoseconds.*” The following year, Zewail won the Nobel Prize in Chemistry for his pioneering work on femtosecond spectroscopy, and some twenty-four years after that, in 2023, the Nobel Prize in Physics was awarded to Agostini, Krausz, and L'Huillier^3^ for their development of groundbreaking experimental methods to generate and exploit attosecond pulses of light.

In parallel with these advances in pushing temporal resolution to its limits, there have been exciting and influential breakthroughs in marrying the ultrafast regime with scanning probe methods – namely, scanning tunnelling microscopy (STM) and atomic force microscopy (AFM).^4–13^ This combination of ultrahigh temporal precision with spatial resolution down to not only the atomic/molecular level, but the single chemical bond regime, now enables condensed matter to be probed (and manipulated) on spatiotemporal scales that even as recently as a few decades ago seemed out of reach. As the theme of this special issue captures, *ultrafast* and *ultrasmall* are now mutually accessible, rather than wholly distinct, domains.

Instead of tracking (sub-)molecular dynamics on ever-faster timescales, however, sometimes it is equally, or more, instructive to reduce the rate of the process(es) of interest. Slower timescales can often be particularly beneficial if the analysis of the system is carried out in the energy domain *via* spectroscopy of some description: energy and time are conjugate Fourier variables and thus related by the uncertainty principle,††Disregarding the well-worn debate about the role of time in quantum mechanics (*i.e.* as a parameter rather than a self-adjoint operator) and the consequent contrast with the position-momentum uncertainty principle. Δ*E*Δ*t* ∼ ℏ, or, slightly more formally, *Γ* ∼ ℏ/*τ*, where *Γ* is the imaginary part of the complex-valued self-energy and *τ* is the lifetime. Extending the characteristic timescale for a process, *i.e.* increasing *τ*, therefore yields a narrowing of spectroscopic peaks in the energy domain, providing better resolved spectra and, thus, greater insights into the underlying dynamics. To secure longer lifetimes, the degree of coupling between the atom/molecule of interest and the environment – which, to choose a particularly pertinent case in point, could be the quasi-continuum of electronic states close to the Fermi level of a metal substrate – must be reduced.

As a key example from an STM perspective, Repp *et al.*^14^ introduced the use of thin NaCl layers as a decoupling layer on metal surfaces, enabling adsorption states that were much closer to the “native” gas phase character of the adsorbate of interest.^14,15^ Extended Hückel theory calculations indicated that, in the absence of coupling to phonons, the lifetime of the highest occupied molecular orbital (HOMO) and lowest unoccupied molecular orbital (LUMO) of a decoupled pentacene molecule on NaCl/Cu(111) is of the order of picoseconds, corresponding in the energy domain to a Lorentzian width of order a few hundred μeV, as compared to the femtosecond timescales (and peak-widths in the hundreds of meV to ∼eV range) characteristic of adsorption on bare metal substrates. Coupling to phonons, however, significantly altered the tunnelling spectrum from this ideal “decoupled” limit – a theme to which we will repeatedly return below.

Although an elegant and widely adopted solution, ultrathin NaCl layers represent just one route to molecular decoupling. Ho and co-workers^16–21^ have long employed thin aluminium oxide films on a metallic substrate as a platform for vibronic spectroscopy of a variety of molecules, including phthalocyanines, porphines, and, of particular relevance to the work described herein, C_60_.^20,21^ As Nazin, Wu, and Ho compellingly demonstrated,^19^ the combination of a thin oxide with the controllable vacuum gap of the STM enables fine control of the relative rates of tunnelling on and off a molecule under the tip, and, consequently, determines the intensity of vibronic features observed in differential conductance, 
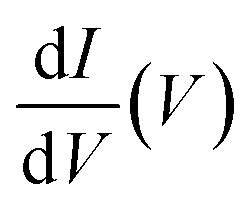
, spectra (and their derivative, 
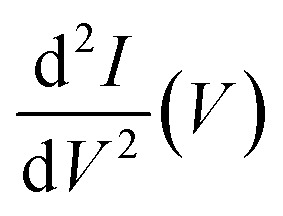
). An increasingly wide range of other substrates and strategies have been adopted for vibronic spectroscopy of this type (and for related techniques such as STM-induced electroluminescence, phosphorescence, and fluorescence^22–27^), including hexagonal boron nitride (h-BN),^28^ graphene,^29^ MoS_2_,^30^ SiC,^31^ molecular multilayers,^24,25^ co-adsorption of other “spacer” molecules,^32^ and ingenious molecular design and synthesis to incorporate native decoupling.^33^ (A relatively recent special issue of the Beilstein Journal of Nanotechnology, *Towards physical and electronic decoupling of organic molecules*,^34^ covers a number of other approaches.)

This type of decoupling strategy is of course not limited to tunnelling spectroscopy at relatively low energies (*i.e.*, within a few eV of the Fermi level) but can also be exploited at much higher energy, including, as we discuss at length below, photoemission and Auger–Meitner techniques using core excitation in the vacuum ultraviolet/soft X-ray regime. As reviewed in depth by Brühwiler *et al.*,^35^ relatively weak adsorbate-substrate coupling is central to the core hole clock technique^36–38^ – an energy-domain variant of ultrafast spectroscopy that enables the rate of ultrafast electron transfer to be determined on timescales ranging from attoseconds^39,40^ to tens of femtoseconds.^41^

In this paper for the *Ultrafast Meets Ultrasmall* special issue, we combine scanning tunnelling microscopy/spectroscopy (STM/STS), X-ray absorption, photoemission, the core hole clock technique, and density functional theory (DFT) calculations to gain insights into the extent of (de)coupling – and, thus, the time scales for electron tunnelling and transfer – for the noble gas endofullerenes Ar@C_60_ and Kr@C_60_ (and the parent C_60_ molecule) adsorbed on Pb surfaces. If we consider the extent of electronic/vibronic coupling in molecular (and supramolecular) systems as spanning a spectrum from weak to strong, a noble gas endofullerene would appear to be very much at the weak-interaction limit: an inert atom encapsulated inside a closed electronic shell. As such, we might expect minimal perturbation of the encapsulate's gas phase structure and, in turn, narrow spectroscopic peaks.

Our choice of Pb as a substrate material was very much motivated by Franke *et al.*'s intriguing observation^42,43^ that Pb(111) interacts with C_60_ in a markedly different manner than other metal surfaces when it comes to vibrational and vibronic spectroscopy: as compared to other low index metal surfaces, only Pb(111) allows for measurement of inelastic tunnelling spectra that resolve all eight Jahn–Teller-active H_g_ modes.^42–45^ By comparison, on Cu(111) the inelastic tunnelling spectrum is entirely flat and featureless,^42,43^ whereas on Ag(110) only the H_g_(*ω*_2_) Jahn–Teller active mode is observed.^46^ Pb(111) substrates thus offer, in principle, the potential for highly sensitive spectroscopic discrimination of (endo)fullerene molecules, not achievable on other surfaces.

Due to the lack of mixing of the orbitals of the encapsulated noble gas with the surrounding fullerene cage (within the energy range accessible by STM),^47–49^ STS probes what might be described as “extramolecular” charge transfer rates, *i.e.* tunnelling of an electron that transiently occupies an orbital of the fullerene cage and couples, to a greater or lesser extent, to the molecular environment, without, as we shall show, any influence of the encaged species (at least on the smallest energy scales accessed in the experiments described here, *i.e.* ∼1 meV). The core hole clock technique, on the other hand, is sensitive to the intramolecular environment of the encapsulated atom: when tuned to an X-ray absorption resonance of the encapsulate, core hole clock spectroscopy provides a measure of the time scale for a photoexcited electron to tunnel away from the intra-cage excitation site. We find that STS, including inelastic tunnelling spectroscopy (IETS), cannot distinguish between empty and filled fullerene cages (in line with our previous work on H_2_O@C_60_ (ref. 50)) suggesting that the encapsulated atom is completely decoupled from its host cage. Core-level spectroscopies, however, instead provide clear evidence of a strong influence of the Pb substrate on the encapsulate despite the apparent decoupling provided by the surrounding cage. We aim here to reconcile these seemingly conflicting observations.

## 2 Methods

### 2.1 Sample preparation

Ar@C_60_ and Kr@C_60_ were synthesised^51,52^*via* the process commonly known as molecular surgery,^53^ enabling a yield of higher than 99% pure endofullerene material. Both endofullerene molecules and the empty, parent C_60_ cage were deposited under ultrahigh vacuum conditions *via* sublimation at temperatures between 620 K and 680 K.

We use two types of Pb(111) substrate. For the STM, STS, and IETS experiments (see immediately following sub-section), thin (111)-oriented Pb films of sub-10 nm thickness were epitaxially grown on a Cu(111) substrate, prepared *via* conventional sputter-anneal cycles, *via* a FOCUS GmbH EFM-3 e-beam evaporator. A Pb(111) single crystal (MaTeck GmbH), cleaned *via* mild Ar^+^ sputter-anneal cycles (500 eV ions, <500 K) was used for the synchrotron-based experiments; the surface cleanliness and crystalline order were checked *via* low energy electron diffraction (LEED) and photoemission survey spectra, ensuring the absence of contaminant core-level peaks (including, in particular, C 1s and O 1s) and a Pb(111) valence band spectrum that showed the characteristic surface resonances previously discussed in the literature.^54–56^ As discussed in the Results section, the thin, epitaxially grown Pb(111) films behaved identically to a Pb(111) single crystal when it came to their interaction with (endo)fullerenes (within the limits of our experimental resolution).

### 2.2 STM, STS, IETS

All scanning tunnelling microscopy/spectroscopy measurements were carried out using a Unisoku USM-1300 STM/qPlus AFM system operating in ultrahigh vacuum (base pressure in the low 10^−10^ mbar range) at 4.2 K controlled by Nanonis Mimea™ electronics and software. Bias voltages are defined with respect to the sample, *i.e.* a positive bias produces an empty states image. The Nanonis digital lock-in amplifier module was used to acquire d*I*/d*V* and d^2^*I*/d*V*^2^ spectra with modulation amplitudes ranging from 2 mV to 20 mV (as specified in the relevant figure captions below). We note that thermal broadening sets a fundamental resolution limit of 5.4*k*_B_*T* (ref. 57) for a normal metal (*i.e.* non-superconducting) tip. Tips were treated by indentation into the substrate (at a bias voltage of between 1 V and 5 V), ensuring initially that the surface state of Cu(111) was well-resolved and, following Pb deposition, that the spectral signatures of the quantum well states of the thin Pb film were comparable to those previously reported in the literature.^58,59^ We note that the thickness of the Pb film is such that the proximity of the Cu(111) substrate reduces the superconducting *T*_c_ to below the experimental temperature of 4.2 K. (Franke *et al.*^42^ have, in any case, carefully checked that operation above or below the critical temperature for superconductivity of bulk Pb (*i.e.* 7.2 K) did not affect their IETS data for C_60_ on single crystal Pb(111)).

### 2.3 Synchrotron-based spectroscopy

Photoelectron spectroscopy (PES), X-ray absorption spectroscopy (XAS), and resonant Auger–Meitner/core hole clock spectroscopy were performed at Beamline I09 of the Diamond Light Source^60^ using a soft X-ray undulator and plane grating monochromator with a resolving power of ∼10 000. Photoelectron peak energies were referenced to the Fermi level of the Pb(111) single crystal substrate. To circumvent beam damage, the undulator was detuned so as to reduce the flux by an order of magnitude; the sample was also cooled to 180 K continuously throughout the experiment (including during endofullerene deposition).

### 2.4 Computational methods

Density Functional Theory (DFT) calculations were performed using the Vienna *Ab initio* Simulation Package (VASP).^61–63^ Exchange–correlation effects were treated within the generalized gradient approximation (GGA) employing the Perdew–Burke–Ernzerhof (PBE) functional.^64^ To accurately describe non-covalent interactions, the DFT-D3 dispersion correction developed by Grimme *et al.*^65^ was incorporated. For the calculation of the density of states (DOS), the SCAN-rVV10 meta-GGA functional was exclusively employed to mitigate inherent limitations associated with the PBE functional in this context.^66^ We employed a plane-wave basis set with a 500 eV cutoff energy, achieving a total energy convergence threshold of 10^−6^ eV. A vacuum spacing of 20 Å along the *c*-axis was employed to eliminate interactions between periodic slab images. The diffusion barrier for Kr@C_60_ was calculated using the climbing image nudged elastic band (CI-NEB) method.^67^ Eleven intermediate images were generated to simulate the diffusion path. Calculations were carried out using the SCAN-D3 approach, with relaxed convergence criteria set to 0.05 eV per Å per atom. Post-processing and detailed analysis of VASP outputs were conducted using VASPKIT.^68^

## 3 Results

**Fig. 1:**
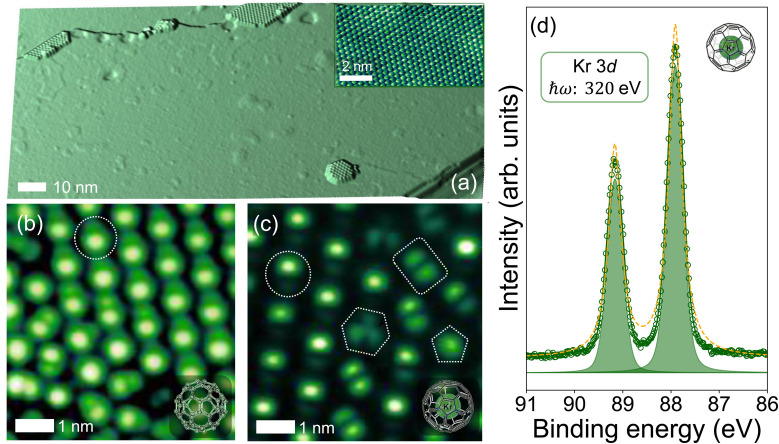
(a) Contrast-enhanced STM image (*V*_b_: −0.51 V, *I*_sp_: 18 pA), rendered with simulated illumination, of a dilute coverage of Kr@C_60_ on a thin Pb film on Cu(111). Inset: Atomic resolution image of the lattice of the Pb film – the lattice constant is identical, within experimental uncertainty, to that of bulk Pb(111): 3.49 ± 0.04 Å (*V*_b_: +1.06 V, *I*_sp_: 20 pA). (b and c) STM data showing intramolecular contrast for C_60_ (constant current image, *V*_b_: −0.3 V, *I*_sp_: 350 pA) and Kr@C_60_ (constant height d*I*/d*V* map acquired at a bias of −10 mV and a modulation amplitude of 10 mV, *i.e.* probing the density of states close to *E*_F_), respectively. Molecules in an atom-down, double-bond-down, hexagon-down, and pentagon-down adsorption state are highlighted by a dotted circle, dotted rectangle, dotted hexagon, and dotted pentagon, respectively. (d) Open green circles: Kr 3d core-level photoelectron spectrum for Kr@C_60_ measured with a photon energy of 320 eV. Solid green line and shaded peaks: fit to the measured spectrum using two Voigt components, representing the spin–orbit split 3d_3/2_ and 3d_5/2_ levels. Orange dashed line: fit using just Lorentzian functions. A linear, almost constant, background has been subtracted.

Deposition of a submonolayer coverage of Kr@C_60_ onto a thin Pb film epitaxially grown on Cu(111) results in a low density of small, well-ordered molecular islands pinned at steps edges or defects (Fig. 1(a)). (The (111) orientation of the Pb film is clear from the atomic resolution image of the lattice shown as the inset to Fig. 1(a): the spacing of the maxima is 3.49 ± 0.04 Å, as compared to the 3.50 Å lattice constant of Pb(111).) STM imaging with submolecular resolution, Fig. 1(b) and (c), reveals no detectable differences between Kr@C_60_ and empty C_60_; a comparison of hundreds of images of empty and filled fullerenes (containing thousands of molecules) shows that STM (in traditional imaging mode) is incapable of discriminating between C_60_ and its endofullerene sibling (as we also found previously for H_2_O@C_60_ (ref. 50)). This is unsurprising given the lack of Kr-derived density of states in the energy window that is accessible to STM (see Fig. 3(b) and ref. 48).

### 3.1 Spectroscopic probes inside and outside the cage

**Fig. 2:**
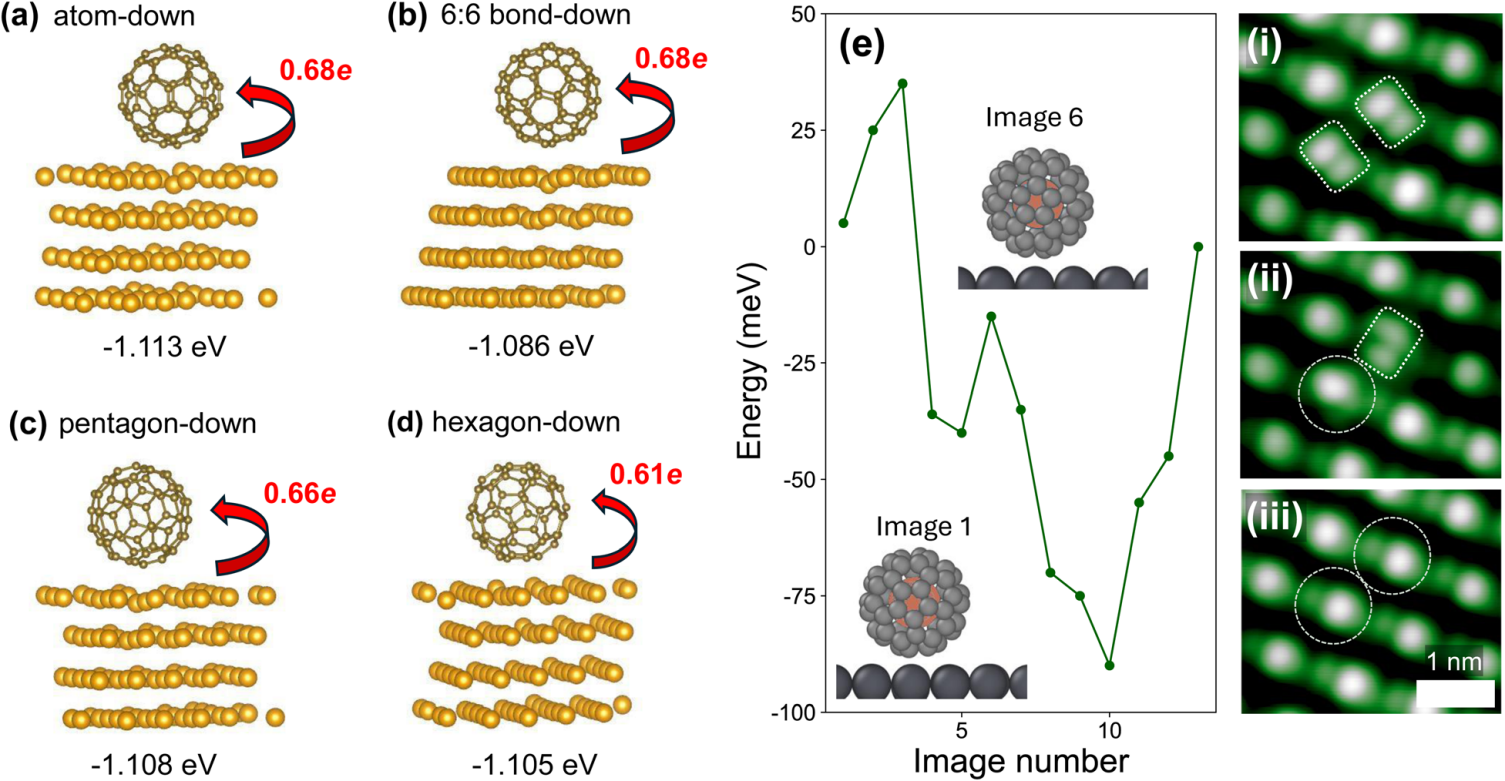
(a–d) Lowest energy adsorption orientations for C_60_ on Pb(111). The atom-down geometry in (a) yields the lowest energy state, although the 6:6 bond-down, hexagon-down, and pentagon-down orientations have very similar adsorption energies. The level of charge transfer from the Pb(111) surface into the fullerene, as predicted by DFT, varies very little as a function of molecular orientation. Note that the addition of Kr inside the fullerene cage does not modify the adsorption energies. (e) Nudged elastic band (NEB) estimation of the rotational barrier for Kr@C_60_ on Pb(111). (See Methods for details of the NEB calculation.) (e(i)–(iii)) STM images (*V*_b_: 0.1 V, *I*_sp_: 1 nA) showing orientational changes of Kr@C_60_ following STS measurements involving sample bias voltages between −0.2 V and 2.3 V and a stabilisation tunnel current of 1 nA.

Submolecular resolution STM images of fullerenes acquired at a negative sample bias of hundreds of mV, or lower, as in Fig. 1, highlight the pentagonal rings (due to the partially filled LUMO near the Fermi level)).^69,70^ We follow Néel *et al.*^71^ and Jung *et al.*^29^ in our interpretation of the variations in molecular contrast seen in Fig. 1(b) and (c) whereby the majority circled features are due to an atom-down adsorption geometry. (See also the discussion below related to Fig. 2.) We find that C_60_ and Kr@C_60_ both have a propensity for an ”atom-down” molecular orientation, with other minority orientations observed less frequently. (Moreover, in many cases these (apparently metastable) orientations are produced *via* perturbation due to the tip; see discussion below re. Fig. 2(e).) From an extra-molecular perspective, therefore, a filled and an empty C_60_ molecule not only yield indistinguishable STM images, but behave in a markedly similar fashion in terms of their interaction with the underlying Pb(111) surface. A clear inference can thus be drawn from the STM data: the encapsulated atom is exceptionally weakly coupled to the cage, and therefore has minimal interaction, at most, with the molecular environment.

Core spectroscopy, however, provides an alternative, and rather different, intramolecular perspective. Although the Kr 3d core-level spectrum for a thick, bulk-like film of Kr@C_60_ (Fig. 1(d)) comprises a pair of well-resolved spin–orbit split peaks (*i.e.* 3d_3/2_, 3d_5/2_) with no evidence for other core-level shifted components, the width of each of the spectral peaks is more than a factor of four larger than observed for gas phase krypton: 380 ± 20 meV *vs.* 88 meV.^72^ Lorentzian functions alone (orange dashed line in Fig. 1(d)) yield a poor fit to the Kr 3d spectrum. A convolution of Lorentzian and Gaussian profiles, *i.e.* a Voigt lineshape, is instead required to provide a good fit (solid green line and shaded peaks). Notably, the broadening is strongly Gaussian in character – the Lorentzian contribution to the overall width of the fitted Voigt functions, 98 ± 5 meV, is very similar to that of the gas phase spectrum. (We should note, however, that the strong Gaussian character of the Voigt components makes the robust extraction of a reliable Lorentzian width somewhat difficult. The error bar reflects only the uncertainty returned *via* diagonalisation of the fit covariance matrix.)

This significant difference in broadening arises from the inhomogeneity in the potential landscape for photoemission^73^ and its influence on both the initial and final state of the photoemission process; for one, Kr@C_60_ molecules at the surface of the film “see” a different electrostatic environment to those in the bulk of the film. (The kinetic energy of the outgoing photoelectrons is ∼200 eV, which equates to an escape depth of less than 1 nm, *i.e.* less than the van der Waals diameter of the endofullerene.) It would appear that the Kr core-level signal essentially acts as a tracer of the potential variations experienced by the parent cage and, as such, the encaged atom is clearly not especially well decoupled from its environment during photoemission. We return to this point below in the discussion of the core hole clock measurements.

### 3.2 DFT: adsorption energies and molecular orientation

A total of 48 distinct geometries for C_60_ on Pb(111) were investigated using DFT, reflecting the broad configuration space arising from the molecule's high symmetry and its wide range of possible polar and azimuthal orientations. (See the SI file for the complete set of geometries/orientations.) The calculations predict that the atom-down geometry (Fig. 2(a)) is the lowest energy adsorption state, in good agreement with the prevalence of this orientation observed in experiment (Fig. 1(b) and (c)). Although the 6:6-bond-down geometry is appreciably higher in energy (by 27 meV; note that *kT* at 4.2 K is ∼360 μeV), other metastable states lie much closer in energy: the hexagon-down (Fig. 2(c)) and pentagon-down (Fig. 2(d)) orientations are only 5 meV and 8 meV less energetically favourable, respectively. Perhaps surprisingly, the amount of charge transfer into the (endo)fullerene molecule from the Pb(111) surface predicted by DFT is essentially independent of molecular orientation: one might expect that a geometry that optimises the overlap of empty state density with the Pb(111) surface (such as the pentagon-down configuration) would facilitate a higher degree of electron donation into the fullerene. However, we reiterate that the energy differences between the various orientations are relatively small as compared to adsorption on other metal surfaces. Moreover, the introduction of Kr inside the fullerene cage did not modify the adsorption energies for the geometries shown in Fig. 2(a)–(d).

Both during imaging and following tunnelling spectroscopy measurements we would sometimes observe spontaneous rotation of C_60_ or Kr@C_60_ molecules; examples are shown in Fig. 2(e(i)–(iii)) for the STS-induced case. In order to determine the barrier for rotation (albeit for a single, isolated molecule), the nudged elastic band (NEB) method^74^ was used. As shown in Fig. 2(e), the barriers predicted by the NEB calculations are of order tens of meV to ∼100 meV. By comparison, during the STS measurements tunnelling electrons with energies up to 2 eV are injected into a (weakly coupled) molecule. In contrast with Néel *et al.*'s tip-driven C_60_ rotation on Cu(100),^71^ we find that the currents required for orientational changes of fullerenes on Pb(111) are orders of magnitude smaller (∼nA as compared to μA).

Given the small relative energy differences arising from fullerene–Pb(111) binding, intermolecular interactions will also make a significant contribution to the overall stability of the molecular assembly. For C_60_ adsorbed on graphene/Cu(111), Jung *et al.*^29^ explain the strong preference for an atom-down orientation they observe as arising from intermolecular interactions that tilt the molecule slightly away from a pentagon-down orientation. It is notable that for C_60_/Pb(111) (and Kr@C_60_/Pb(111)), the atom-down geometry is (weakly) preferred even in the absence of intermolecular interactions. Jung *et al.* also observed fullerene rotation on graphene during imaging under relatively “mild” tip-sample interaction conditions (*viz.*, a sample bias of +1.5 V and tunnel current of 50 pA), suggestive of comparably weak fullerene–substrate interactions for graphene and Pb(111).

### 3.3 The dynamic Jahn–Teller effect

Due to their unique symmetry and, thus, highly degenerate electronic and vibrational states, C_60_ and endofullerenes (including Ar@C_60_ and Kr@C_60_) are particularly prone to Jahn–Teller (J–T) distortions in an ionic or excited state.^75–78^ In tunneling spectroscopy experiments with C_60_ molecules that are weakly coupled to their environment, transient charging of the molecule can lead to coupling between the electronic state and JT-active molecular vibrations. When the timescale of the tunneling event is long enough for nuclear motion to respond, vibronic coupling can lead to observable Jahn–Teller distortions. A distortion that remains localized in a single symmetry-broken minimum is referred to as a static Jahn–Teller effect. In contrast, if the molecule explores multiple equivalent minima—either through quantum tunneling or classical thermal barrier-crossing—the distortion is considered dynamic.

A particularly relevant example of the dynamic JT (DJ–T) effect in the context of our work is that of Frederiksen *et al.*,^32^ who co-deposited C_60_ and 1,3,5,7-tetraphenyladamantane (TPA) on Au(111), producing a supramolecular assembly where the fullerene molecules were raised away from the metal substrate by TPA. Sharp sidebands were subsequently observed in tunnelling spectra of the LUMO of the substrate-decoupled C_60_, which Frederiksen *et al.*, *via* an insightful analysis informed by previous theoretical calculations by Manini *et al.*^79^ and Gunnarsson *et al.*,^80^ attributed to the DJ–T effect. They applied both Manini *et al.*'s^79^ approach, *i.e.* exact diagonalization of the electron-vibration Hamiltonian (*via* the Lanczos scheme), and non-equilibrium Green's function techniques to determine the J–T-induced fine structure in the local density of states of the LUMO.

**Fig. 3:**
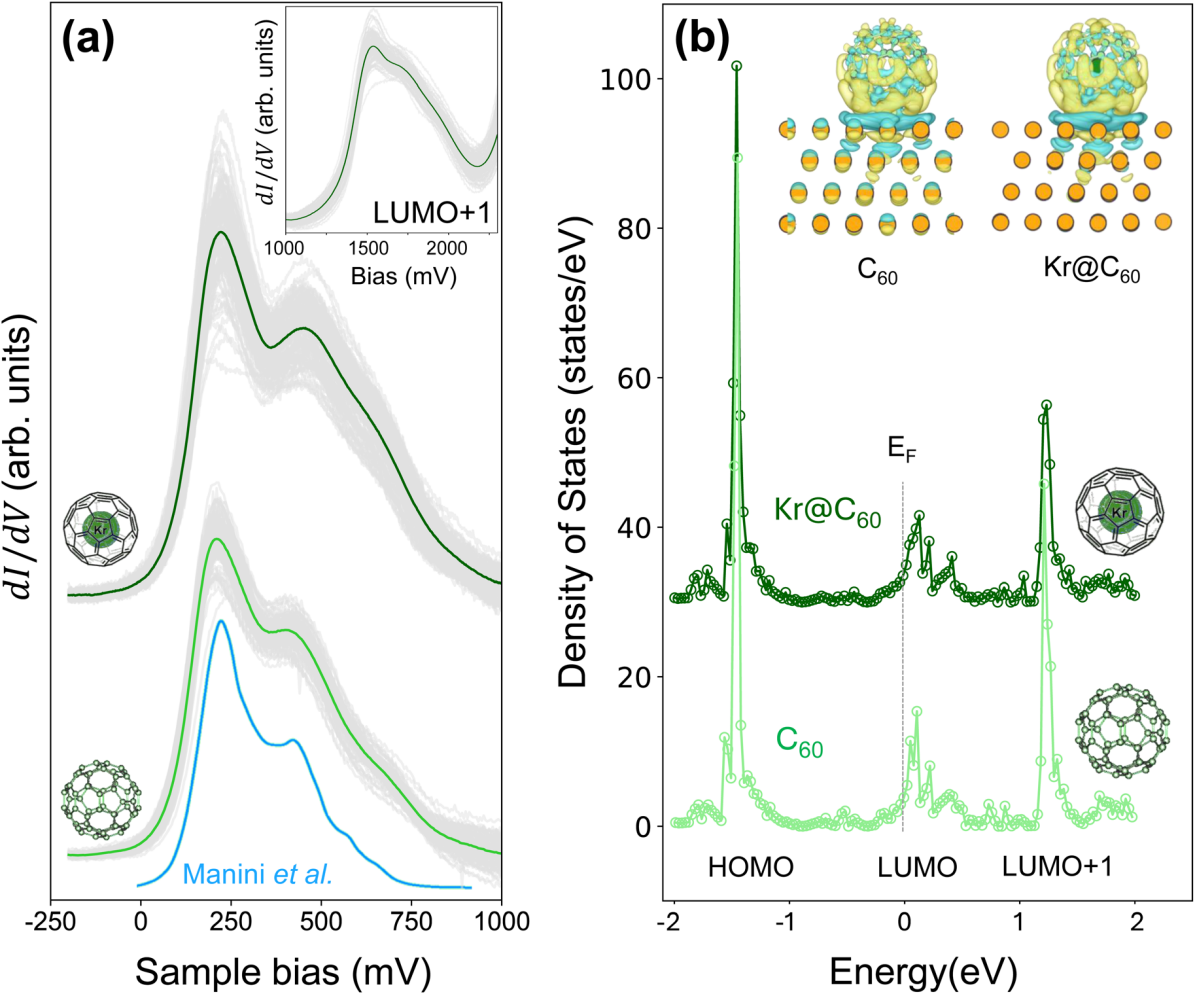
(a) Tunnelling spectra of the LUMO-derived empty states density for (lower) empty C_60_ and (upper) Kr@C_60_ adsorbed on a thin (111)-oriented Pb film on Cu(111). The Fermi level is located at 0 bias. Grey curves: a set of 58 (for C_60_) and 81 (for Kr@C_60_) spectra acquired in the central region of close-packed fullerene islands to illustrate variation in spectral lineshape from molecule to molecule. Solid green and dark green lines: average spectrum for C_60_ and Kr@C_60_, respectively. Blue solid line: Calculated spectrum of the h_u_ hole spectral intensity function for C_60_ HOMO photoemission that has been digitally traced from Manini *et al.*,^79^ and rigidly shifted along the sample bias axis so that the peak intensity coincides with that of the measured tunnelling spectra. (b) Density of states between −2 eV and + 2 eV calculated for (lower) C_60_/Pb(111) and (upper) Kr@C_60_/Pb(111) using the SCAN meta-GGA functional (see Computational methods). Inset: Charge density difference maps for C_60_ and Kr@C_60_ on a Pb(111) substrate, calculated using the SCAN meta-GGA functional (see Computational methods). Cyan isosurfaces indicate charge depletion, while yellow isosurfaces represent charge accumulation.

Frederiksen *et al.*'s calculations for the C_60_–TPA/Au(111) system reproduced a central result of Manini *et al.*'s^79^ first principles study for the free C_60_ molecule: the appearance of a vibronic sideband at an energy, ∼230 meV, that is significantly greater than that of the highest frequency Jahn–Teller-active vibrational mode, *i.e.* H_g_(*ω*_8_) ∼ 195 meV. Indeed, Manini *et al.*'s calculated spectrum, which we have digitally traced and reproduced at the bottom of Fig. 3(a) for comparison and convenience, features a second shoulder that is energetically distant from the LUMO resonance by as much as 400 meV. (We note that a weak peak appearing approximately 400 meV from the LUMO maximum is also visible in the LUMO spectrum published by Frederiksen *et al.*^32^)

A visual comparison of the measured LUMO lineshape (for both C_60_ and Kr@C_60_) with Manini *et al.*'s calculated spectrum (Fig. 3(a)) reveals a notable resemblance, suggesting a possible common underlying DJ–T mechanism. Although similarities in spectral lineshapes do not, of course, represent incontrovertible evidence of a common vibronic origin‡‡Moreover, Manini *et al.*'s^79^ simulated spectrum is essentially derived from the h_u_ ⊗ H_g_ direct product (the G_g_ and A_g_ vibrational modes do not make a strong contribution) in order to model photoemission spectra of the C_60_ HOMO, whereas DJ–T-derived fine structure in the STS LUMO spectra is a t_1u_ ⊗ H_g_ JT problem. (We use uppercase (lowercase) letters to represent vibrational modes (electronic states).) While the ungerade–gerade parity constraints are met in each case, it should be noted that the degeneracies of the HOMO and LUMO are different (five-fold *vs.* three-fold, respectively). we provide additional support below for the role of the DJ–T effect in modulating the LUMO lineshape (in relation to molecules that are more effectively decoupled from the substrate *via* a spacer layer). In addition, we note that a very similar ∼200 meV splitting of the LUMO (and higher energy shoulder) has been observed in tunnelling spectra of C_60_ on a variety of weakly interacting surfaces including single-crystal Pb(111),^42–45^ graphene,^29^ and, most recently, SnSe(001).^81^

Although the LUMO spectrum for Kr@C_60_ shown in Fig. 3(a) (dark green line) is broader than that for C_60_, we have observed comparable variations in broadening simply as a function of molecular orientation and environment for each class of molecule (*i.e.* empty or filled). As such, the apparent additional broadening for Kr@C_60_ is not related to the presence of endohedral Kr but simply reflects variations in molecular orientation and/or environment. (See also the discussion re. inelastic tunnelling spectra in the following sub-section.) A high degree of inhomogeneous broadening of LUMO spectra due to the orientation and/or environment of adsorbed C_60_ has also been reported by Meierott *et al.*^44,45^ for adsorption on Pb(111) and by Bommert *et al.* in their study of fullerene adsorption and charging on hBN/Rh(111).^28^

### 3.4 Comparison of tunnelling spectra with DFT-predicted DOS

To explain the much greater efficacy of Pb(111) as a substrate for IETS of adsorbed C_60_, Franke *et al.*^42,43^ have suggested that a subtle balance of two factors may be key: a sufficiently high density of states (DOS) at the Fermi level should be accompanied by a low level of damping of molecular vibrations (*i.e.* long vibrational state lifetimes) *via* electron–hole pair creation. With regard to the density of states at *E*_F_, tunnelling spectra for both C_60_ and Kr@C_60_ on a thin (111)-oriented Pb film on Cu(111) (Fig. 3(a)) are in good agreement with those of both Franke *et al.*^42,43^ and Meierott *et al.*^44,45^ for adsorption on a Pb(111) single crystal: the low-energy tail of the LUMO extends to, and slightly beyond, the Fermi level, resulting in a small but finite density of states at zero bias.

Our DFT calculations for C_60_ and Kr@C_60_ on a Pb(111) slab (Fig. 3(b)) reproduce, for both molecules, the small density of states at *E*_F_ seen in experiment. Although the DFT calculations exhibit some indication of splitting within the LUMO-derived density of states, the overall width of the LUMO spectrum is rather narrower than that observed experimentally. If we accept that the LUMO lineshape is indeed a consequence of the DJ–T effect, then this observation is unsurprising, given that conventional DFT operates within the Born–Oppenheimer approximation and does not account for vibronic coupling of the dynamic Jahn–Teller form. We note also that the experimentally observed splitting of the LUMO+1 spectral peak (inset to Fig. 3(a)) is not reproduced in the DFT calculations; again, if the LUMO+1 splitting were due to the DJ–T effect then this is to be expected.§§Any broadening due to LUMO band formation in the solid state^82^ is significantly lower than that observed in the tunnelling spectra.

Inset to Fig. 3(b) are charge density difference plots for both C_60_ and Kr@C_60_ on Pb(111) that show the presence of an interface dipole in each case. Incorporation of krypton inside the cage does not affect the magnitude of the interface dipole nor the spatial distribution of the charge density. Furthermore, and as previously observed for the Ar@C_60_ endofullerene on Ag(111),^49^ in its ground state the encapsulated atom is not influenced by the formation of the cage-Pb(111) interface dipole. In contrast, and as discussed in detail below, the influence of the underlying Pb(111) surface on the encapsulate is clearly seen in photoemission spectra *via*, for one, the influence of final state screening.

### 3.5 Can IETS detect caged atoms?

**Fig. 4:**
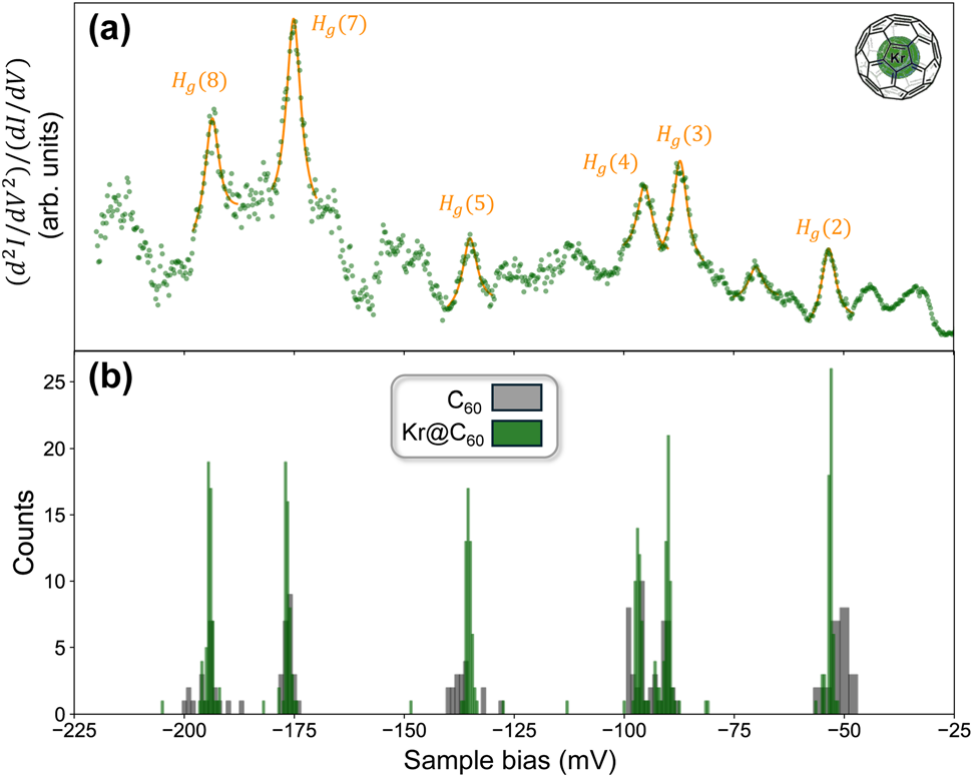
(a) Inelastic tunnelling spectrum for a Kr@C_60_ molecule on a (111)-oriented Pb thin film on Cu(111) acquired with setpoint stabilisation conditions of −225 mV, 50 nA and a lock-in amplifier modulation amplitude of 2 mV. The d^2^*I*/d*V*^2^ spectrum has been divided by the first derivative, d*I*/d*V* in order to reduce the spectral background. To further reduce the background contribution we also focus here on the negative bias side of the spectrum – see Fig. 3(a) to compare the d*I*/d*V* spectral character in the ±200 mV range. We have inverted the spectrum so that the inelastic features are peaks rather than dips. Six of the Jahn–Teller active H_g_ modes of the fullerene cage are highlighted in orange and have been fit with a Lorentzian lineshape. Although other peaks are visible, as discussed in the body of the paper we see significant variation in the IET spectra depending on molecular orientation, environment, and the position of the tip with regard to nodes and antinodes of the LUMO state density. As such, we focus on the most commonly and reliably observed peaks in both positive and negative bias. (b) Histogram of energies of H_g_(2), H_g_(3), H_g_(4), H_g_(5), H_g_(7), and H_g_(8) modes for C_60_ (dark grey) and Kr@C_60_ (green) molecules on a thin (111)-oriented Pb film on Cu(111). Within the limits of our experimental resolution, there is no difference in the energies of the modes for filled *vs.* empty fullerene cages.

As noted in the Introduction, a core motivation for our IETS measurements of C_60_ and endofullerenes on Pb(111) was to determine whether tunnelling-based vibrational spectroscopy, *i.e.* IETS, could distinguish between empty and filled cages *via* slight shifts in the energies of the JT-active modes.^42,43^ Our selection of Kr@C_60_ was on the basis of the slightly larger blue-shift (as compared to other noble gas endofullerenes) – up to ∼2 meV – observed for the infra-red vibrational bands^83^ in relation to those of the empty parent molecule. Fig. 4 shows both a representative IETS spectrum for Kr@C_60_ (Fig. 4(a)) and a statistical comparison of the energies of the most prominent JT-active modes observed across all spectra, namely H_g_(*ω*_2_), H_g_(*ω*_3_), H_g_(*ω*_4_), H_g_(*ω*_5_), H_g_(*ω*_7_), H_g_(*ω*_8_) (or H_g_(*n*) for short).

Across all spectra, the H_g_(7) mode is the most intense feature in the Kr@C_60_ IET spectra, in line with not only Franke *et al.*'s^42^ (and our own) IETS measurements for empty C_60_ but the higher level of electron–phonon coupling predicted for this mode by many first principles calculations.^79,80,84^ We stress, however, that we see considerable variation in the intensity of the H_g_ modes depending not only on molecular orientation and environment (*e.g.* number of nearest neighbours, proximity to a step edge), as previously observed by Meierott *et al.*,^44,45^ but also on the precise placement of the tip on submolecular scales: for some molecules, tunneling into a node of the LUMO density can completely “quench” one or more of the H_g_ modes seen in Fig. 4 as compared to an IET spectrum attained above an antinode of the same molecule. This effect, and its connection to fullerene orientation, symmetry, and electron–phonon coupling will be the focus of a separate future publication.

In addition, lower temperature (∼340 mK), and thus much higher resolution, IETS measurements of endofullerenes are planned. On this point, we note that Jafari *et al.*^85^ have observed an intense peak at ∼11 meV in THz absorption spectra (acquired at ∼5 K) of Ar@C_60_, and a somewhat broader feature (due to the contribution of different isotopes) centred at ∼10 meV for Kr@C_60_. In each case, the absorption signal arises from the response of the C_60_-induced dipole moment of the encapsulated noble gas atom to the incident THz radiation. For the reasons discussed above in relation to encapsulate-cage coupling (and the lack thereof) in the tunnelling process, our expectation is that IETS at 340 mK – notwithstanding its much higher, sub-meV resolution – will not reveal the mode detected by THz spectroscopy.

### 3.6 Native decoupling: vibronic ladders and DJ–T modulation

**Fig. 5:**
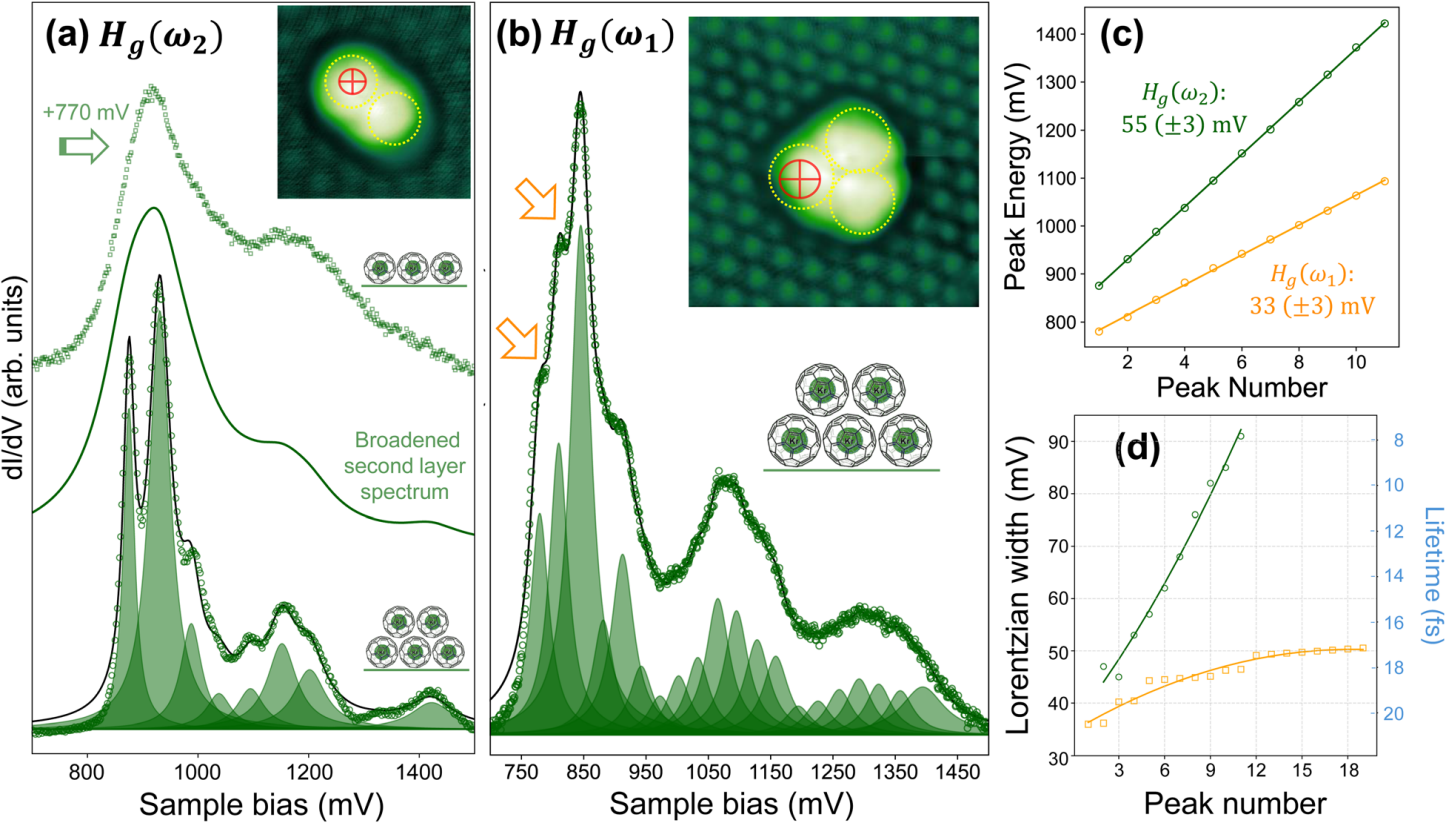
Vibronic progressions in the LUMO spectra of electronically decoupled Kr@C_60_ molecules. Inset: Contrast-enhanced STM image (*V*_b_: 3.5 V, *I*_sp_:10 pA) of a second layer Kr@C_60_ vdW “dimer”. (Dotted circles are simply guides to the eye.) The crosshairs (in red) represent the tip position for measurement of the LUMO spectrum described in the following. (a) H_g_(*ω*_2_) (∼55 meV) progression. Lowermost spectrum: open circles: measured d*I*/d*V* LUMO spectrum; solid line: multi-Lorentzian (shaded peaks) fit to the measured spectrum (without background removal) where the peak separation is constrained, within ±3 meV, to the H_g_(*ω*_2_) energy of 55 meV. The Lorentzian peak width increases with sample bias (see (d) and body of paper); central spectrum: broadened version of fit to second layer spectrum, where the Lorentzian width of each peak has been increased to 120 meV; uppermost spectrum: d*I*/d*V* spectrum of LUMO resonance measured for a first layer Kr@C_60_ molecule, offset by +770 mV in order to align the spectral peak with that for the second layer molecule. (b) H_g_(*ω*_1_) (∼33 meV) progression. The arrows point to sharp vibronic features that are offset by one H_g_(*ω*_1_) quantum or two H_g_(*ω*_1_) quanta, respectively, from the highest intensity resonance. The inset is an STM image (*V*_b_: 2.5 V, *I*_sp_: 10 pA) of the Kr@C_60_ vdW “trimer” for which the measurement was made; the red crosshairs again represent the tip position. (Again, a background has not been included in the fit.) (c) Energy of the Lorentzian components of the fit *vs.* peak number; (d) width of the Lorentzian components *vs.* peak number. The width has been converted to a lifetime (right axis) *via* the energy-time uncertainty relation. Lines are guides to the eye.

To slow the rate of electron tunnelling off a fullerene molecule still further, we use the first Kr@C_60_ layer as a “native” decoupling interface, in a similar manner to the strategy employed by Große *et al.*^25^ for electroluminescence mapping of thin fullerene films. Fig. 5(a) and (b) show examples of vibronic spectra acquired in this way for a Kr@C_60_ van der Waals (vdW) “dimer” and vdW “trimer”.¶¶Square quotes are used to denote that, distinct from dimers and trimers found in covalently bonded fullerene species (see, for example, Cardillo-Zallo *et al.*^86^), here we refer to van der Waals-bound clusters. Tunnelling spectra of second layer molecules are significantly more structured than those of their first-layer counterparts (which interact directly with the underlying Pb surface), in line with the increased lifetime, and thus narrower linewidth, of the transient ionic state of the decoupled endofullerene. As Pradhan *et al.*^20,21^ first observed for C_60_ molecules decoupled from their substrate *via* an ultrathin oxide film, the reduction in linewidth enables vibronic peaks to be observed directly in the differential conductance, d*I*/d*V*, spectrum itself. In this resonant tunnelling regime, the linewidth due to the lifetime of the tunnelling process is smaller than the energy of the particular vibrational quantum (or quanta) underpinning the vibronic coupling.^43^

In the lowermost spectrum of Fig. 5(a) the open circles represent the measured d*I*/d*V* spectrum acquired at the point highlighted by the red “crosshairs” in the image shown in the inset. This spectrum can be captured to a reasonably good first approximation by a series of Lorentzian peaks spaced by 55 ± 3 meV, *i.e.* the energy quantum associated with the H_g_(2) mode. The leading edge of the spectrum (onset ∼800 meV) is, however, not at all well-described by the low energy side of the first Lorentzian in the series. We believe that the sudden onset of the spectrum arises from the abrupt shift in LUMO energy due to field-induced charge transfer, as observed for other electronically decoupled molecules;^87^ a symmetric Lorentzian lineshape therefore cannot account for the spectral profile arising from the initial charge transfer event.

While, as noted above, progressions of the type seen in Fig. 5 have previously been observed for the parent C_60_ molecule,^20,21^ it is the overall modulation of the LUMO lineshape that is of particular interest in the context of tunnel rates and vibronic coupling in decoupled (endo)fullerene molecules. We include in Fig. 5(a) (uppermost spectrum) the LUMO profile acquired for a first layer Kr@C_60_ molecule in the vicinity of the vdW dimer (blue-shifted in energy by 770 meV), which displays the signature form of the lineshape that we have tentatively attributed to the DJ–T effect (previous section). If we take the fit to the second layer LUMO spectrum and increase the full-width-at-half-maximum (FWHM) of each constituent Lorentzian peak to a value of 120 meV, this broadened spectrum (also shown in Fig. 5(a)) bears comparison with the first layer lineshape, suggesting that the H_g_(2)-derived vibronic progression may be modulated by the DJ–T effect. For a vdW trimer (Fig. 5(b)) we see the same overall modulation of the LUMO spectrum as for the vdW dimer, with broad “satellite” peak structure offset from the primary resonance by, again, ∼+230 meV. In this case, however, the vibronic progression is accounted for by a series of Lorentzian peaks with a 33 ± 3 meV separation, *i.e.* the H_g_(1), rather than the H_g_(2), quantum. (Note that the peak separation is constrained to ± 3 meV in the fit for both Fig. 5(a) and (b).||||Given the large parameter space, the fit is not unique and we are acutely aware of von Neumann's famous maxim: “with four parameters I can fit an elephant, and with five I can make him wiggle his trunk.”^88^) As was also observed for Fig. 5(a), and for what we suggest is the same reason, the abrupt onset of the spectrum is not well captured by the leading Lorentzian peak.

In Fig. 5(c) we plot the fitted energies of the Lorentzian peaks comprising the spectra – the strong linearity is not especially surprising given that the fit is constrained to allow only ±3 meV variation in the positions. Less immediately obvious, however, although nonetheless apparent in the raw data, is that the linewidth of the Lorentzian peaks increases – or, correspondingly, the lifetime of the vibronic state decreases – with the energy of the tunnelling electrons above the Fermi level (Fig. 5(d)). A similar broadening as a function of energy has been reported very recently by Lou *et al.*^81^ for C_60_ adsorbed on SnSe(001), also for a vibronic progression arising from the H_g_(1) mode. However, our measurements of the H_g_(1)-governed progression differ from those of Lou *et al.* in that, as is clear from Fig. 5(a) – and in line with Pradhan *et al.*'s work involving a thin oxide decoupling layer^20,21^ – the fine structure in the LUMO lineshape does not exclusively arise from the lowest energy Jahn–Teller mode.

We also stress that, as noted above for the first monolayer, the spectra vary dramatically with placement of the tip on a submolecular scale. We suggest that the observation of the H_g_(2) *vs.* the H_g_(1) mode in Fig. 5(a)*vs.*Fig. 5(b) arises from both the difference in the molecular environment and the precise tunnelling “injection” point inside the molecule due to tip placement. Unfortunately, it was not possible to achieve submolecular resolution for second (and higher) layer endofullerenes bound as isolated molecules, “dimers” or “trimers”; tip-molecule interaction forces invariably resulted in molecular translation due to the closer proximity of the tip required to attain internal molecular contrast.

Observation of the H_g_(1) mode energy of ∼33 meV is also notable in the context of the dielectric properties of the Kr@C_60_ decoupling layer. In principle, the measured energy for a given Jahn–Teller mode, H_g_(*n*), should require a correction due to the finite voltage drop in the endofullerene layer: *E*_meas._ = (1 − *α*)*E*_H_g_(*n*)_, where a rough estimate for *α* can be made on the basis of a highly simplified parallel plate capacitor model, *α* = *d*/(*d* + *εz*), where *d* is the thickness of the fullerene layer, *ε* is the fullerene dielectric constant, and *z* is the tip-sample separation.^89^ Assuming a value of *d* of 1 nm (the vdW diameter of C_60_; the atom-to-atom diameter is 0.71 nm), a tip-sample separation of 0.5 nm, and a value of *ε* of 4, we estimate *α* ∼ 0.3. This is a substantial correction factor that is, however, not required to account for the separation of the vibronic peaks in Fig. 5. (The arrows in Fig. 5(b) highlight well resolved features offset from the main LUMO peak by H_g_(1) quanta.) Even if we were to assume a 1 nm tip-sample separation as an upper limit, the correction factor is still appreciable, *α* ∼ 0.2, and leads to a bias correction that is significantly outside the ±3 mV uncertainty of the separation in Fig. 5(b). However, as discussed above, the endofullerene layer is weakly doped by charge transfer from the underlying Pb, meaning that use of the dielectric constant of undoped fullerite to determine *α* likely represents a significant underestimate of *ε*.

### 3.7 Endofullerenes on Pb(111): PES and XAS

We now turn to synchrotron-based spectroscopies and measurement of intramolecular charge transfer/tunnelling rates *via* the core hole clock (CHC) technique. In order to apply the CHC protocol, and for reasons explained in the Clocking intramolecular charge transfer sub-section below, we shifted our focus to the Ar@C_60_ molecule. Nonetheless, although the synchrotron-based measurements were conducted on a different noble gas endofullerene, the STM and tunnelling spectroscopy data presented in earlier sections demonstrate that the frontier electronic structure and spectral signatures of noble gas endofullerenes are indistinguishable from those of empty C_60_. This close similarity provides confidence that insights from the tunnelling studies can be meaningfully related to the X-ray spectroscopies, despite the use of a different endohedral species.

**Fig. 6:**
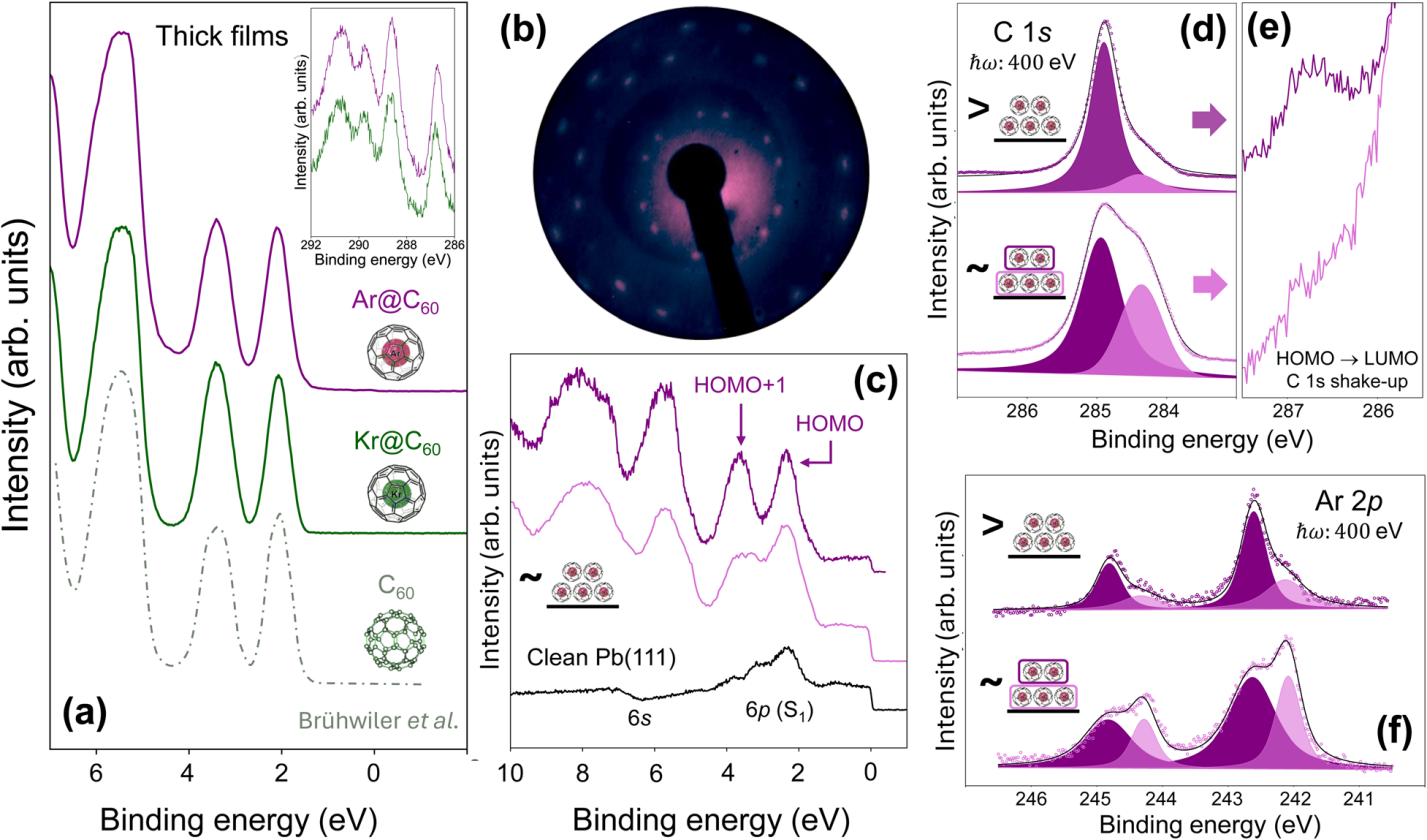
Valence band and core-level spectroscopy for endofullerenes on Pb(111). (a) Valence band photoelectron spectra, each acquired with a photon energy of 110 eV, for bulk-like films of Ar@C_60_, Kr@C_60_, and C_60_ (digitally traced from Brühwiler *et al.*^90^). Inset: C 1s shake-up spectra for Kr@C_60_ (green) and Ar@C_60_ (purple). The lowest energy peak is due to a HOMO → LUMO transition; (b) low energy electron diffraction (LEED) pattern (beam energy: 27 eV, acquired at 180 K) for Ar@C_60_/Pb(111); (c) valence band spectra (again acquired at a photon energy of 110 eV) for the clean Pb(111) surface (lowermost spectrum in black) and for two different coverages of Ar@C_60_. The spectrum shown in light purple is for the endofullerene coverage that produced the LEED pattern shown in (b). The 6s- and 6p-derived surface resonances for Pb(111) are highlighted. The latter was originally labelled as the S_1_ resonance by Würde *et al.*;^55^ (d) C 1s spectra for the different Ar@C_60_ coverages giving rise to the VB spectra shown in (c). The core-level shifted component shaded in light purple in each case is due to the first layer Ar@C_60_, adsorbed directly on the Pb(111) surface. (e) C 1s HOMO → LUMO shake-up region for the thin and thick Ar@C_60_ film. (f) Corresponding Ar 2p spectra. As for (d), the core-level-shifted component arising from the first Ar@C_60_ layer is shaded in a light purple colour.

Additional experimental support for the electronic similarity – at least for energies within a few eV of the Fermi level – of the noble gas endofullerenes is provided by Fig. 6(a), in which valence band spectra acquired at a photon energy of 110 eV (the lowest available at beamline I09) for thick films of Ar@C_60_ and Kr@C_60_ are plotted alongside a spectrum for empty C_60_ (also acquired with ℏ*ω* = 110 eV) that has been digitally traced from Brühwiler *et al.*^90^ In each case, the highest occupied molecular orbital (HOMO)-derived feature at ∼2.2 eV and the HOMO+1 peak at ∼3.5 eV exhibit indistinguishable binding energies and linewidths.**There is a difference in the background of the Ar@C_60_ and Kr@C_60_ spectra (most noticeable in the spectral region between the HOMO+1 peak and the next highest energy feature at ∼5.8 eV) arising from a slight difference in film thickness. More interestingly, while the background for the Kr@C_60_ film is comparable to that of the empty C_60_ spectrum,^90^ the relative intensities of the HOMO and HOMO+1 peaks are reversed for Kr@C_60_ as compared to C_60_. A likely origin of this effect is photoelectron interference inside the fullerene cage.^91^ Similarly, the C 1s shake-up spectra shown in the inset to Fig. 6(a) for both Kr@C_60_ and Ar@C_60_, and which arise from a set of transitions between HOMO and LUMO states,^92^ bear a close resemblance. Although the shake-up peaks with binding energies above 288 eV are due to a complicated manifold of transitions between different filled and empty states of the fullerene cage, the feature at 286.7 eV, *i.e.* offset from the primary C 1s peak by 1.8 eV, has a much more straight-forward explanation: it originates in a HOMO → LUMO (*i.e.*
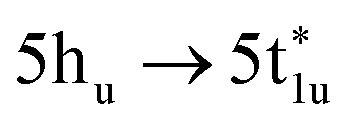
) transition. That the HOMO–LUMO shake-up feature in particular is essentially independent of the noble gas species again highlights the lack of influence of the encapsulate on the electronic structure of the cage.

Given the unusual nature of the Pb(111) substrate from the perspective of IETS,^42,43^ we have carried out valence band/core-level PES and Ar L_3_ edge XAS measurements in order to gain further insights into the extent of molecule–substrate coupling (and to inform the core hole clock analysis described in the next section). A thin film of Ar@C_60_, whose thickness we estimate as approximately bilayer††††Given that the sample temperature was 180 K during Ar@C_60_ deposition, and that there is an appreciable Schwoebel–Ehrlich barrier associated with diffusion across fullerene step edges,^93^ there is a strong likelihood that the second (and higher) Ar@C_60_ layer(s) starts to form before the first completes. was deposited onto the clean Pb(111) surface resulting in the low energy electron diffraction (LEED) pattern shown in Fig. 6(b). This pattern is essentially the same as that previously reported by Li *et al.*^94^ for C_60_/Pb(111), albeit with poorer spot definition and a higher background due, we suggest, to the lower temperature (180 K) at which the Ar@C_60_ was deposited (thereby restricting both translational and rotational fullerene motion, yielding smaller domain sizes).

A valence band photoemission spectrum of the clean Pb(111) surface before endofullerene deposition, acquired with a photon energy of 110 eV (lowermost spectrum (in black) in Fig. 6(c)), displays the characteristic 6p- and 6s-derived resonances^54–56^ and a sharp Fermi edge feature. Deposition of Ar@C_60_ onto the Pb(111) surface yields the signature fullerene valence band features (upper spectra (in shades of purple) in Fig. 6(c)), although there is strong overlap between the HOMO and HOMO+1 states and the 6p-derived S_1_ surface resonance,^54^ which spans binding energies between 2 eV and 4 eV. Unlike the STS measurements and DFT calculations shown in Fig. 3, the valence band spectra for Ar@C_60_/Pb(111) do not provide clear evidence for fullerene-derived density of states at the Fermi level. This is unsurprising: not only is the Fermi level DOS measured by tunnelling spectroscopy and predicted by DFT rather weak (Fig. 3), but the photon energy of 110 eV is far from optimal in terms of optimising photoabsorption cross-section for emission from carbon-derived valence states.

C 1s core-level photoemission data (Fig. 6(d)) corresponding to the valence band spectra shown in Fig. 6(c) comprise two components: one, shaded in light purple, arises from the Ar@C_60_ molecules in the first layer that directly interact with the Pb(111) surface, and the other (dark purple) from molecules in higher layers. The first-layer C 1s component is shifted by 600 ± 10 meV towards lower binding energy (as compared to the bulk Ar@C_60_ peak) due to enhanced screening by the Pb(111) substrate – a similar 600 meV shift has been observed for C_60_ adsorption on Cu(111).^95^ In principle, the core-level component arising from the first layer could – arguably, should – be fit with a Doniach–Sunjic (D–S) lineshape to capture the influence of the metallic screening of the core-hole. However, we found that the C 1s spectrum could be equally well fit with a Voigt lineshape for the first-layer component – the overlap with the symmetric bulk component makes discerning the screening-derived asymmetry difficult – and thus there is little direct justification in the data for the inclusion of a D–S profile. (In contrast, for adsorption of Ar@C_60_ on Ag(111), a Doniach–Sunjic lineshape provided a better fit.^49^ See also discussion below re. the Ar 2p core-level lineshape.)

As the thickness of the Ar@C_60_ layer increases, and the film approaches the bulk limit, the FWHM of the higher binding energy C 1s component narrows. In parallel, the HOMO → LUMO shake-up feature, which is entirely washed out for the lowest Ar@C_60_ coverage investigated, increases in intensity above the background (Fig. 6(e)). This coverage-dependent evolution of the C 1s spectrum for Ar@C_60_/Pb(111) is not only entirely in line with our previous results for Ar@C_60_/Ag(111)^49^ but is characteristic of the behaviour of the parent C_60_ molecule on a variety of metal surfaces.

Thus far, photoemission—like the tunnelling spectroscopy measurements of Fig. 3–5—has primarily probed the fullerene cage and the ‘extra-molecular’ surroundings. For the remainder of this paper we focus on Ar-derived photoemission, X-ray absorption, and Auger–Meitner spectra, which provide insights into the intramolecular environment and associated dynamics. A key result, which again echoes our analysis of the Ar@C_60_/Ag(111) system,^49^ is the observation that the Ar 2p core-level spectrum (Fig. 6(f)) exhibits an almost identical core-level shift (*i.e.* relative to the bulk-derived peak) for photoemission from the first layer molecules as the C 1s level: 550 ± 30 meV. In other words, the encapsulated atom, far from being entirely decoupled as the tunnelling spectroscopy measurements would suggest, clearly “senses” the molecular environment, including, in particular, the screening due to the Pb(111) substrate. Indeed, we found that a better fit of the Ar 2p spectrum (Fig. 6(f)) resulted when the first-layer component was modelled as a Doniach–Sunjic, rather than a Voigt, lineshape. Moreover, the narrowing of the bulk-derived core-level component seen for the C 1s spectrum is also observed for the Ar 2p data.

### 3.8 Clocking intramolecular charge transfer

**Fig. 7:**
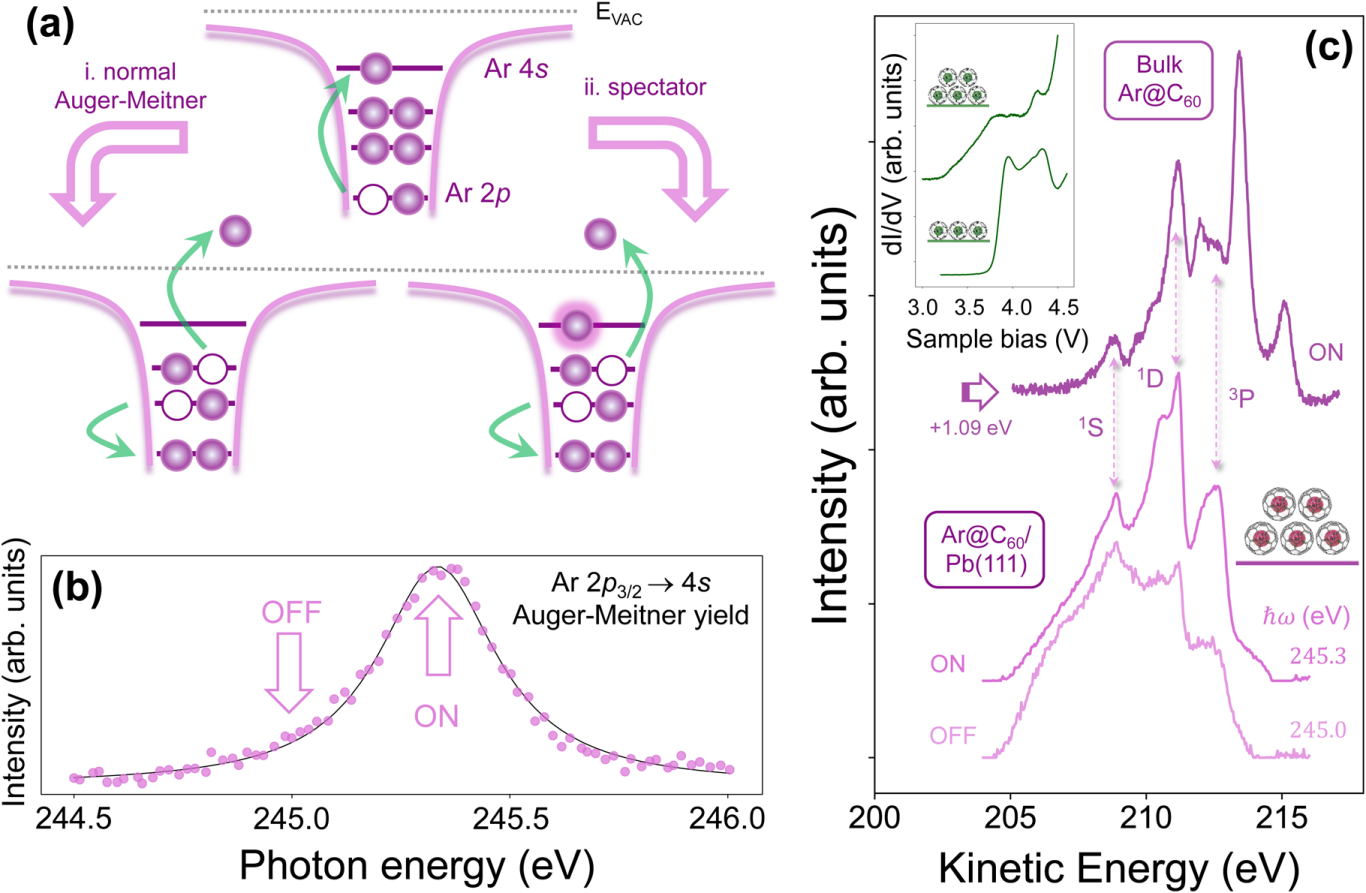
Core hole clock/resonant Auger–Meitner spectroscopy of Ar@C_60_/Pb(111). (a) Schematic illustration of the primary processes in the CHC technique. Resonant X-ray excitation (upper diagram) is followed by core-hole decay either *via* (i) normal Auger–Meitner emission (where the photoexcited 4s electron has tunnelled away from the excitation site before the core hole decays), or (ii) spectator decay, where the 4s electron delocalisation time is longer than the core hole lifetime. (b) Ar 2p_3/2_ → 4s X-ray absorption spectrum for the approximate bilayer Ar@C_60_ film that gave rise to the LEED pattern (Fig. 6(b)) and associated valence band and core-level spectra shown in Fig. 6; (c) Auger–Meitner decay spectra for (lower) the approximate bilayer Ar@C_60_/Pb(111) sample both on and off resonance, and (upper) a bulk Ar@C_60_ film. The inset shows tunnelling spectra for the superatomic orbital resonances of (lower) first layer and (upper) second layer Kr@C_60_ molecules.

Despite lacking the high level of temporal control possible with time-domain pump–probe techniques, with a judicious choice of system (and energy levels), core hole clock spectroscopy can determine electron delocalisation times far into the attosecond regime.^38–40^ With this in mind, we had initially hoped to exploit the super-Coster–Kronig, *i.e.* very rapid, decay of the Kr 3p core-excited state^96^ to track electron delocalisation in Kr@C_60_ (for direct comparison with the STM and STS data discussed in previous sections). A short-lived state, however, translates to a broad resonance in the conjugate energy domain. When coupled with the high background signal due to the Pb(111) substrate (see below in relation to Fig. 7), the lower effective quality factor for the resonance essentially ruled out CHC spectroscopy at the Kr 3p (*i.e.* M_2,3_) edge.‡‡‡‡Although the Kr 3d level for Kr@C_60_ is well resolved (Fig. 1), and its lifetime width is ∼90 meV, its binding energy of ∼88 eV falls below the lower energy limit of beamline I09 (*i.e.* 110 eV), precluding resonant excitation.

We therefore turned to the Ar@C_60_ endofullerene as a probe of intramolecular delocalisation dynamics. For the reasons discussed by Menzel^38^ and Brühwiler *et al.*,^35^ argon is particularly well-suited for CHC spectroscopy. Moreover, we have recently carried out a detailed CHC study of Ar@C_60_ on Ag(111);^49^ as discussed in the following, the Ar@C_60_/Pb(111) system bears many similarities to Ar@C_60_/Ag(111).

Fig. 7(a) schematically highlights the key processes underlying CHC spectroscopy at the Ar L_3_ edge. Following resonant X-ray excitation from the 2p_3/2_ level to the originally empty 4s state, there are two primary non-radiative channels for decay of the core-excited state: so-called normal Auger–Meitner decay (process (i) in Fig. 7(a)), and spectator decay (process (ii)). For the former, the electron excited to the 4s level has tunnelled away from the excitation site before the core hole decays, whereas, for spectator decay, the delocalisation rate of the 4s state is sufficiently slow that core-hole decay happens in the presence of the photoexcited electron. (As described by Field *et al.*,^49^ participator decay plays a negligible role: the intensity of the Ar 3s photoemission peak remains essentially constant as the photon energy is varied across the Ar 2p_3/2_ → 4s resonance. See Fig. 2(c) of ref. 49.)

By sweeping the photon energy, ℏ*ω*, through the Ar 2p_3/2_ → 4s excitation and, in parallel, integrating across the Auger–Meitner/spectator kinetic energy window at each value of ℏ*ω*, an X-ray absorption resonance spectrum (Fig. 7(b)) is acquired. In this case, the spectrum was acquired from the same ∼ bilayer coverage sample from which the C 1s and Ar 2p spectra of Fig. 6 were measured. As compared to a bulk Ar@C_60_ film, the resonance width is higher: 330 (±10) meV *vs.* 280 ± 10 meV. A 330 meV width translates to a ∼2 fs lifetime, assuming that the only contribution to the absorption spectrum is the lifetime width. (The solid line in Fig. 7(b) is a Lorentzian fit to the Ar 2p_3/2_ → 4s resonance.)

Background-subtracted Auger–Meitner decay spectra measured on and off resonance – at photon energies of 245.3 eV and 245.0 eV, respectively – are shown in Fig. 7(c) (lowermost spectra, light purple) for the ∼ bilayer coverage Ar@C_60_/Pb(111) sample. As previously observed by Vijayalakshmi *et al.*^97^ for argon adsorption on Cu(111) and Cu(100) surfaces, there is a broad tail at lower kinetic energies arising from relatively low energy electron–hole excitations due to the metallic substrate. This tail, along with the strong background due to the close proximity of the Pb 5d core-level (which has been removed from the spectra shown in Fig. 7(c)), makes extracting reliable peak intensities *via* fitting (as we have previously carried out for a bulk Ar@C_60_ film^49^) challenging at best. In any case, fitting the spectra would provide little additional information with regard to electron delocalisation times, for the following reasons.

For operation in the resonant Raman regime (as is the case here), where the photon energy bandwidth is much lower than the core hole lifetime broadening,^38,98^ spectator peaks remain at a fixed binding energy as ℏ*ω* is varied; normal Auger–Meitner peaks instead have fixed kinetic energy. A comparison of the on-resonance and off-resonance Ar@C_60_/Pb(111) Auger–Meitner spectra (bottom of Fig. 7(c)) shows that the spectral peaks and shoulders remain at fixed kinetic energy – in other words, there is no evidence for a spectator contribution to the decay process. This lack of spectator contribution, in turn, means that the photoexcited Ar 4s electron has tunnelled away from the initial excitation site before core-hole decay. A direct comparison with the Auger–Meitner spectrum measured for bulk Ar@C_60_, also shown in Fig. 7(c), bears out the lack of spectator contribution: there is no evidence for spectator-shifted peaks but, by shifting the bulk spectrum by 1.09 eV to account for differences in screening, a one-to-one matching of the ^1^S,^1^D, and ^3^P components of the normal Auger–Meitner spectrum can be made.

There is, however, a clear splitting of the central (^1^D) peak in the on-resonance spectrum for the ∼ bilayer sample. We interpret this as a difference in screening of the two-hole Auger–Meitner final state for molecules in the first layer as compared to those in higher layers. However, an additional consideration relates to the density of empty states into which the photoexcited 4s electron can tunnel. We have postulated that the rapid, sub-500-attosecond, delocalization of the Ar 4s electron observed in CHC spectroscopy of the Ar@C_60_/Ag(111) system arises from a mixing of the Ar density with a superatomic orbital (SAMO)^99–102^ of the fullerene cage, which in turn couples to the Ag(111) substrate.

Inset to Fig. 7(c) are tunnelling spectra of SAMO states in the appropriate energy range (albeit for Kr@C_60_ rather than Ar@C_60_) which, intriguingly, and despite a strongly varying background (due to the transmission coefficient), exhibit a much broader tail for the second layer molecules compared to the spectra for the first layer adsorbed directly on the (111)-oriented Pb film. We do not yet understand the origin of this difference but it is nonetheless clear that, in addition to screening-related differences, the density of available empty states may well differ strongly for endofullerenes in different molecular layers. We also note that, as compared to the tunnelling resonances at lower energy (Fig. 5), the SAMO resonances are wide, of order hundreds of meV, and comparable to the width of the Ar 2p_3/2_ → 4s resonance. This higher width, and thus shorter lifetime, is in line with the lower effective barrier height experienced by the electrons tunnelling into the SAMO states.

Given the intense background signal, extracting a credible quantitative value for the characteristic delocalisation time of the Ar 4s electron is, at best, exceptionally problematic. We therefore adopt a much more conservative “heuristic” approach to estimate an upper limit for the delocalisation time. We first take a ratio of the peak intensity of the most intense Auger–Meitner feature (*i.e.* the ^1^D component at ∼211 eV kinetic energy in Fig. 7(c)) and the dominant spectator contribution (^2^D, ∼213.4 eV) in the bulk decay spectrum (Fig. 7(c)) as broadly representative of the spectral weight of the contributions as a whole.§§§§From detailed fitting of the bulk Ar@C_60_ decay spectra,^49^ we know that this simple approach overestimates the charge delocalisation time by about 25%. The absence of any spectator intensity at a kinetic energy of 213.4 eV in the Ar@C_60_ thin film spectrum means that the ^2^D spectator contribution can be, at the very most, comparable to the background intensity at that energy. This means that the highest possible ratio of spectator-to-Auger–Meitner intensity is in turn ∼0.35. This translates to an absolute upper limit of the delocalisation time, *τ*_D_ of 1.7 fs, which is very similar to the ∼2 fs lifetime derived from the width of the Ar 2p XAS resonance alone (Fig. 7(b)). For Ar@C_60_/Ag(111), where the background intensity was much less intrusive, we determined an upper limit of 500 attoseconds^49^ due to the stronger interaction of the fullerene cage with the silver surface.

That tunnelling spectroscopy yields a very different picture of charge transfer as compared to measurements and analyses based on core-level excitation/de-excitation (X-ray absorption, photoemission, and Auger–Meitner relaxation) is unsurprising. The charge transfer pathways (or matrix elements) underpinning d*I*/d*V* and d^2^*I*/d*V*^2^ spectroscopy involve states that are markedly different from those involved in the core-hole-clock approach, not least due to the presence of the core potential for the latter. Fullerenes are an especially good example of the strong perturbation resulting from the core hole. In C K-edge X-ray absorption spectroscopy, the core-hole potential is sufficiently strong so that the energies (and energy separations) of the unoccupied state features in an XAS spectrum bear no similarity to those measured by inverse photoemission (for which the core potential is absent). Similarly, the core potential for the encapsulated atom arising from X-ray excitation will shift its orbitals with respect to those of the fullerene cage. In the absence of the core potential, there is marginal mixing of the encapsulate orbitals with those of the surrounding fullerene, and thus the encaged atom appears “invisible” to traditional STM and STS methods.

## 4 Conclusions

Combining STS and core spectroscopy provides insights into charge transfer and electron delocalisation not only on very different energy/time scales, but from complementary intra- and extra-molecular perspectives. We find that thin (111)-oriented Pb films epitaxially grown on Cu(111) surfaces behave identically to the single crystal Pb(111) surface in terms of enabling the resolution of Jahn–Teller-active (endo)fullerene vibrational modes in IETS. Attributing the lineshape of LUMO tunnelling spectra for first-layer (endo)fullerenes on Pb/Cu(111) to the dynamic Jahn–Teller effect rationalises the modulation observed for second-layer LUMO features. Strong vibronic progressions are also observed for second-layer molecules due to the electronic decoupling provided by the underlying (endo)fullerene layer. The local molecular environment plays a critical role in determining which vibrational mode dominates the vibronic progression, as previously observed for studies involving a thin oxide film as a decoupling layer.^20^

For tunnelling spectroscopy of frontier fullerene orbitals, the encapsulated atom plays an entirely negligible role: STM images and STS measurements of Kr@C_60_ and empty C_60_ are indistinguishable, as expected given the lack of encapsulate state density in the accessible energy window. (In principle, the presence of the encapsulated atom should affect tunnelling spectra of superatomic orbital (SAMO) density but we do not yet have strong experimental evidence to support this supposition.) As such, the encaged atom is essentially perfectly decoupled from the molecular environment.

Core spectroscopies, however, paint a very different picture: Ar 2p photoemission, X-ray absorption, and Auger–Meitner spectroscopy each show that the encapsulated noble gas atom is well coupled to the environment, sensing the presence of the underlying Pb(111) surface. Not only are significant Ar 2p core-level shifts observed for first- *vs.* higher layer endofullerene molecules, but a Doniach–Sunjic lineshape – the signature of metallic screening of the photoexcited state – provides a better fit to the first-layer spectrum. Moreover, the magnitudes of the C 1s and Ar 2p core-level shifts for the first layer (as compared to the corresponding core-level energies for a bulk endofullerite film) are very similar. We reach a similar conclusion as found for Ar@C_60_/Ag(111):^49^ instead of isolating the photoexcited state of the encapsulate from the molecular environment, the cage acts as a conduit for Ar 4s delocalisation, although it appears that the tunnel rate is rather slower for the endofullerene/Pb(111) system due to the weaker coupling to the metal. This longer lifetime may also contribute to the strong enhancement of the Jahn–Teller-active modes seen in IETS of fullerenes on Pb(111) but not other metal surfaces.^42,43^

In future work, we are keen to combine the submolecular resolution of STM and STS with the chemical and intracage specificity of core level spectroscopies *via* the novel STM-XAS approach very recently pioneered by Ajayi *et al.*^103^

## Author contributions

T. J., C. F., F. J., S. A. P., D. A. D., Y. W., B. K., J. N. O. S. and P. M. carried out the experiments during beamtime allocations at I09, Diamond Light Source; A. S., S. N., and J. A. L. were responsible for the DFT calculations; S. B., G. H., M. W., E. S. M., and R. J. W. provided the endofullerene samples; T.-L. L. provided key advice and expertise related to the beamline experiments; C. F., T. J., and P. M. were responsible for experimental data analysis; P. M. drafted the paper, with input and feedback from all co-authors.

## Conflicts of interest

There are no conflicts to declare.

## Supplementary Material

NA-OLF-D5NA00727E-s001

## Data Availability

All raw data for this article – STM images and tunnelling spectra (in Nanonis formats); photoemission, X-ray absorption, and Auger–Meitner spectra (in NeXuS format, https://www.nexusformat.org/) – are available at the University of Nottingham Research Data Management Repository (DOI: https://doi.org/10.17639/nott.7585). Please contact the corresponding author at philip.moriarty@nottingham.ac.uk for Python (Jupyter Notebook) code that opens, plots, and fits (where applicable) these data. Supplementary information: additional density functional theory (DFT) and tunnelling spectroscopy data. See DOI: https://doi.org/10.1039/d5na00727e.
