# Supplementary material for: Charge transfer dynamics in noble gas endofullerenes: intra- and extramolecular tunnelling
Source: Nanoscale Adv. 2025 Oct 10;7(24):7913–29. doi: 10.1039/d5na00727e (PMC12523590; doi:10.1039/d5na00727e)
Supplement: NA-007-D5NA00727E-s001 [file NA-007-D5NA00727E-s001.pdf]

Supplementary Information (SI) for Nanoscale Advances.
This journal is © The Royal Society of Chemistry 2025

Supplementary Information – Charge transfer dynamics in noble gas endofullerenes: intra- & extramolecular tunnelling

Ali Sufyan *et al.*

July 28, 2025

Raw data

Please note that all raw data are available via the University of Nottingham Data Management Repository, DOI: 10.17639/nott.7585.

Supplementary Data – DFT and STS

Table S1: Energy (in eV) of different C_{60}/Pb(111) adsorption sites and molecular rotations.

Configuration	Rotations (deg.)	E (eV)		
		On-top	hcp-hollow	fcc-hollow
atom-down	0	-899.1516	-899.1369	-899.1209
	30	-899.1869	-899.0752	-899.1562
	60	-899.1431	-899.1425	-899.1272
	90	-899.1338	-899.1115	-899.1260
6-6 Bond down	0	-899.1581	-899.1245	-899.1015
	30	-899.1587	-899.1122	-899.1230
	60	-899.1593	-899.0986	-899.1014
	90	-899.1192	-899.1286	-899.1431
pentagon down	0	-899.1241	-899.1401	-899.1473
	30	-899.1361	-899.1212	-899.1051
	60	-899.1297	-899.1260	-899.1441
	90	-899.1812	-899.1026	-899.1190
hexagon down	0	-899.0362	-899.1480	-899.1788
	30	-899.1360	-899.1129	-899.1484
	60	-899.0630	-899.1240	-899.1509
	90	-899.1575	-899.1267	-899.1341

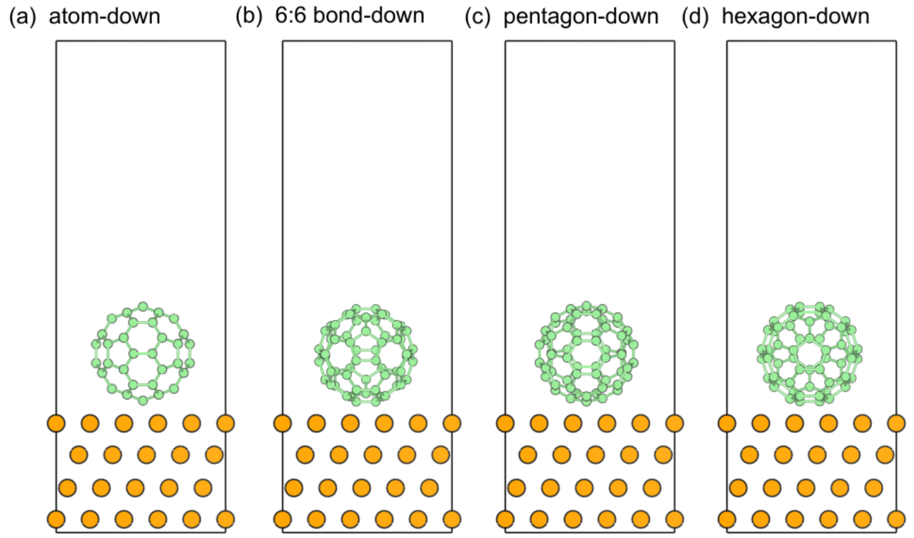

Figure S1: Side view of C_{60} adsorption on the Pb(111) substrate for various orientations: (a) atom-down, (b) 6:6 bond-down, (c) pentagon-down, and (d) hexagon-down.

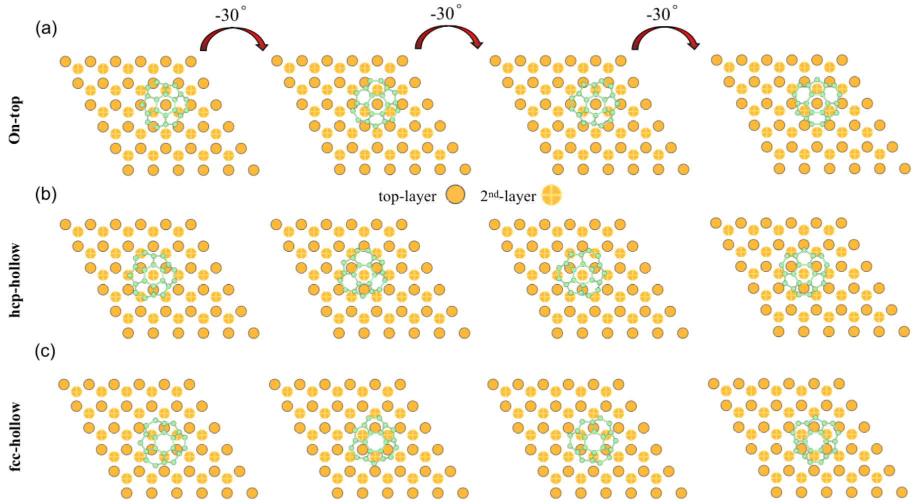

Figure S2: Top view illustrating the atom-down orientation of C_{60} on the Pb(111) substrate for various adsorption sites and rotations. (a) On-top site, (b) hcp-hollow site, and (c) fcc-hollow site. For each site, the C_{60} molecule is shown at rotations of 0°, 30°, 60°, and 90° (from left to right). The orange circles represent Pb atoms (top-layer: orange, 2nd-layer: highlighted orange), and the green circles indicate the lowest C atoms of C_{60} molecule.

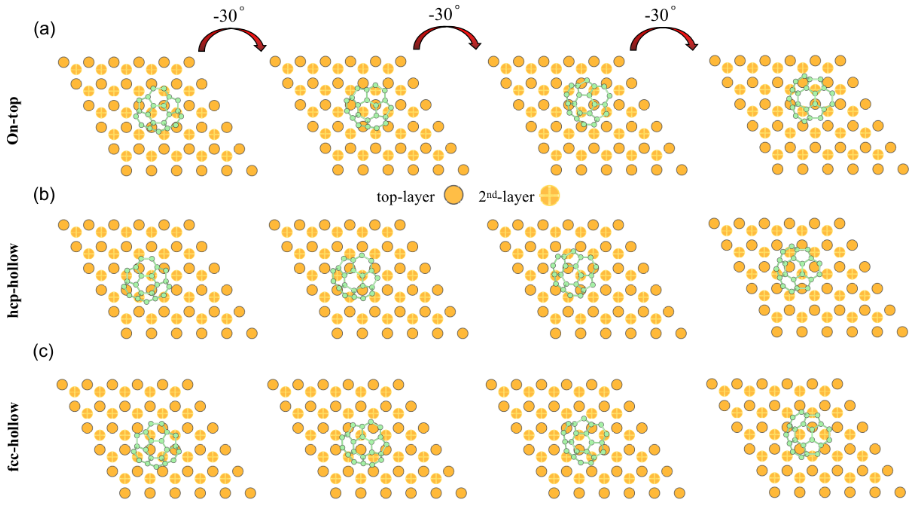

Figure S3: Top view illustrating the 6:6 bond-down orientation of C_{60} on the Pb(111) substrate for various adsorption sites and rotations. (a) On-top site, (b) hcp-hollow site, and (c) fcc-hollow site. For each site, the C_{60} molecule is shown at rotations of 0°, 30°, 60°, and 90° (from left to right). The orange circles represent Pb atoms (top-layer: orange, 2nd-layer: highlighted orange), and the green circles indicate the lowest C atoms of C_{60} molecule.

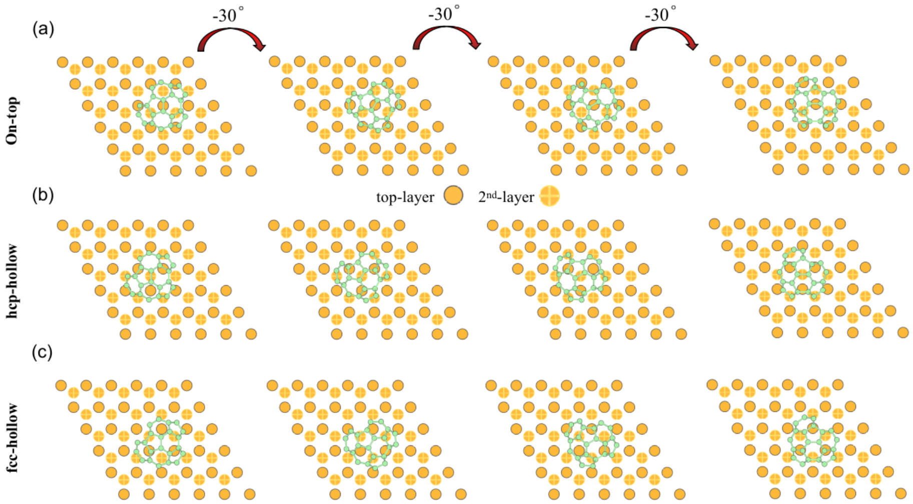

Figure S4: Top view illustrating the pentagon-down orientation of C_{60} on the Pb(111) substrate for various adsorption sites and rotations. (a) On-top site, (b) hcp-hollow site, and (c) fcc-hollow site. For each site, the C_{60} molecule is shown at rotations of 0°, 30°, 60°, and 90° (from left to right). The orange circles represent Pb atoms (top-layer: orange, 2nd-layer: highlighted orange), and the green circles indicate the lowest C atoms of C_{60} molecule.

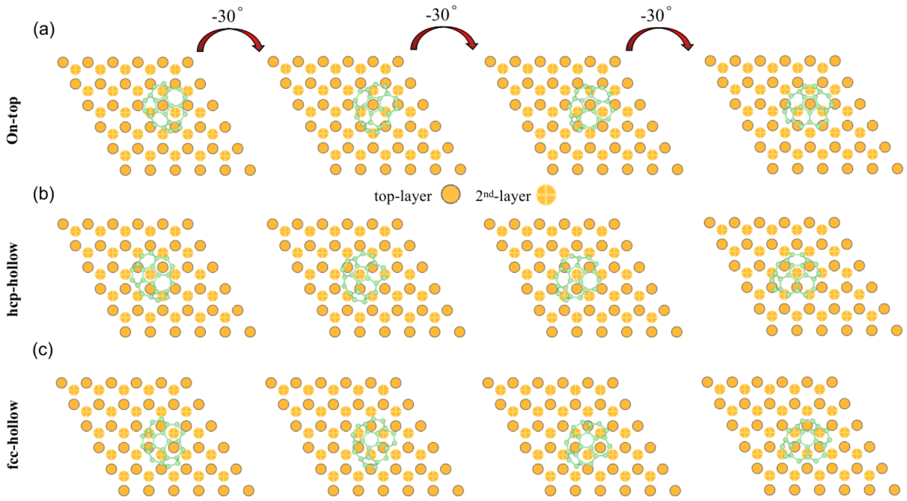

Figure S5: Top view illustrating the hexagon-atom orientation of C_{60} on the Pb(111) substrate for various adsorption sites and rotations. (a) On-top site, (b) hcp-hollow site, and (c) fcc-hollow site. For each site, the C_{60} molecule is shown at rotations of 0°, 30°, 60°, and 90° (from left to right). The orange circles represent Pb atoms (top-layer: orange, 2nd-layer: highlighted orange), and the green circles indicate the lowest C atoms of C_{60} molecule.

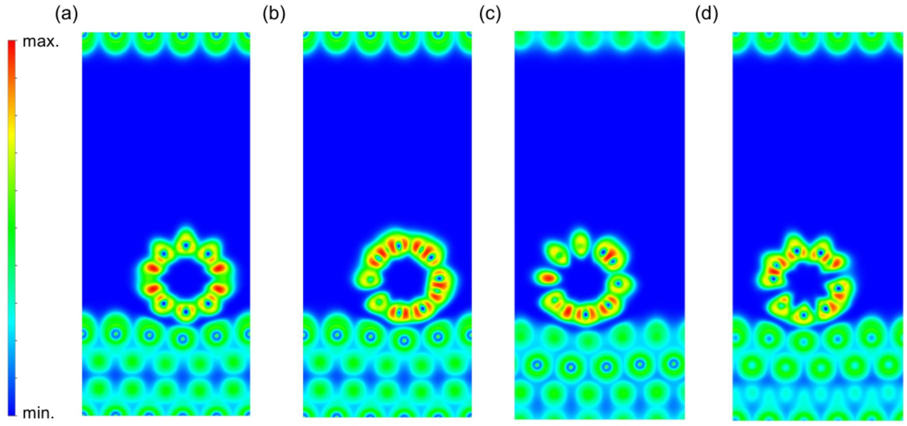

Figure S6: Electron Localization Function (ELF) plots showing the interaction between C_{60} and the Pb(111) substrate for various configurations. (a) Atom-down C_{60} with 30° rotation on an on-top site. (b) 6:6 bond-down C_{60} with 60° rotation on an on-top site. (c) Pentagon-down C_{60} with 90° rotation on an on-top site. (d) Hexagon-down C_{60} with 0° rotation on an fcc-hollow site. The color scale on the left indicates the ELF value, from minimum (blue) to maximum (red).

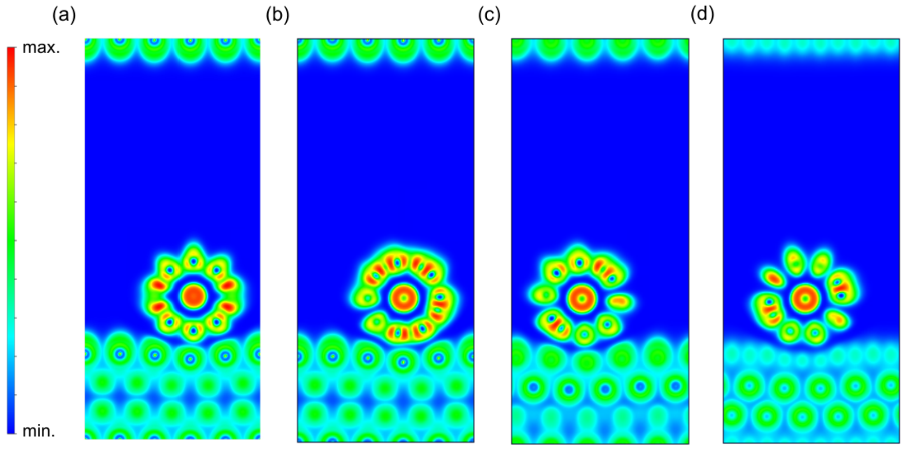

Figure S7: Electron Localization Function (ELF) plots showing the interaction between Kr@C_{60} and the Pb(111) substrate for various configurations. (a) Atom-down Kr@C_{60} with 30° rotation on an on-top site. (b) 6:6 bond-down Kr@C_{60} with 60° rotation on an on-top site. (c) Pentagon-down Kr@C_{60} with 90° rotation on an on-top site. (d) Hexagon-down Kr@C_{60} with 0° rotation on an fcc-hollow site. The color scale on the left indicates the ELF value, from minimum (blue) to maximum (red).

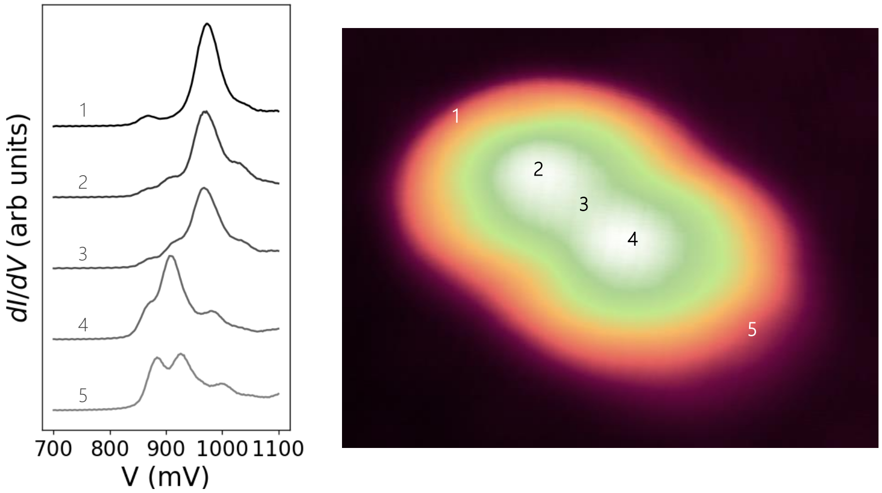

Figure S8: Set of dI/dV spectra measured across a Kr@C_{60} van der Waals dimer adsorbed on top of a complete Kr@C_{60} layer. Note extensive variation in lineshape as a function of tip position.
